# Rheology Indicators for Assessing Bead Spreading of Hydrogels with Functional Rheology Modifiers for Direct Ink Writing: A Case Study for Chitosan–Graphene–Titanium Dioxide

**DOI:** 10.1021/acsapm.5c02887

**Published:** 2025-09-08

**Authors:** Daniel Alves Heinze, Supreet Thale, Yimin Yao, John P. Reynolds, Mark L. Ballentine, Christopher S. Griggs, Christopher B. Williams, Michael J. Bortner

**Affiliations:** † Macromolecules Innovation Institute, Virginia Tech, 240 W Campus Dr, Blacksburg, Virginia 24061, United States; ‡ Department of Chemical Engineering, Virginia Tech, 635 Prices Fork Rd Suite 245, Blacksburg, Virginia 24061, United States; § Department of Mechanical Engineering, Virginia Tech, 445 Goodwin Hall, 635 Prices Fork Road—MC 0238, Blacksburg, Virginia 24061, United States; ∥ US Army DEVCOM Army Research Laboratory, 2800 Powder Mill Road, Adelphi, Maryland 20783-1197, United States; ⊥ Environmental Engineering, 57629 U.S. Army Engineer Research and Development Center—ERDC, 3909 Halls Ferry Rd, Vicksburg, Mississippi 39180, United States

**Keywords:** polymer-matrix suspensions, rheology, 3D printing, particle reinforcement, hydrogels, material extrusion additive manufacturing

## Abstract

The rheological behavior of a model system composed of chitosan (CS) hydrogels with graphene and titanium dioxide (TiO_2_) as functional fillers is studied to define a design space between rheology modification and bead spreading in direct ink writing (DIW) additive manufacturing (AM). Rheological results are combined with printed bead studies to determine how each formulation component affects extrudability and bead shape retention, identifying tan delta as the strongest indicator of good shape retention. Samples with tan delta >1 exhibited ink spreading postdeposition, while those with predominantly solid-like behavior (tan delta ≤ 1 at low angular frequencies) and low-to-intermediate TiO_2_ concentrations extruded well and maintained their shape in the time scale of printing. Further increasing functional particle content (25 wt % TiO_2_) led to inconsistent extrusion that hindered adequate extrusion fidelity. Moreover, the time-dependent structural recovery of the formulations was strongly influenced by composition, with an increasing CS content being less detrimental to storage modulus recovery than the addition of fillers. Finally, the stress required for the inks to transition from a solid-like to a liquid-like state did not correlate with inconsistent extrusion, which were instead linked to high particle concentrations. This work provides insights into the effects of rheology modification using functional particles in DIW AM of hydrogel composites, ultimately helping to improve the efficiency of producing polymer-based hydrogel inks.

## Graphical abstract

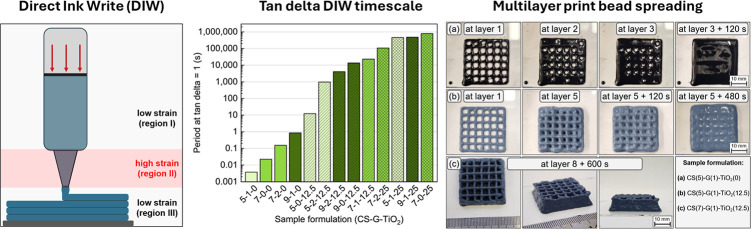

## 1 Introduction

Additive manufacturing (AM) is a growing field of manufacturing in which parts are made using a layer-by-layer approach, allowing the production of unique shapes with an increased surface area.^1^ For polymer composites, one mode of AM of interest is material extrusion (MEX) AM, of which direct ink writing (DIW) or robocasting is a subset, which works by extruding a viscous material (ink) through a nozzle and selectively depositing it layer by layer to form the desired shape. DIW is relevant because it can print a large catalog of materials, including precursor gels and highly filled polymeric systems, as long as the rheological behavior of the ink is appropriately tuned.^1–4^

DIW can be broken down into subfunctions, each with specific rheological considerations,^5^ where commonly explored rheological requirements for DIW include yield stress behavior, adequate viscosity, and shear thinning behavior.^6,7^ The yield stress behavior captures the ability of materials to not flow until a critical stress level is exerted on them, which may be desired to facilitate shape retention of the printed part prior to its solidification. Viscosity represents the resistance to flow of the material, with a shear thinning behavior indicating a decrease in viscosity with increasing shear rates, being important to facilitate flow during material extrusion, where higher shear rates are exerted.^5,8,9^ Higher viscosities at rest (zero shear viscosity) can also contribute to increased resistance to flow of the deposited bead.

When considering bead retention, a rheological indicator of interest for assessing DIW shape retention is tan delta, which describes the ratio between loss (*G*″) and storage moduli (*G*′) of samples. Values of tan delta smaller than 1 indicate a predominant solid-like response (*G*′ > *G*″), while values larger than 1 indicate a predominant viscous-like response (*G*″ > *G*′). Research has shown that values of tan delta between 0.175 and 0.45 were effective for DIW printing of some hydrogels.^6,7^ In addition to these rheological requirements, the thixotropic behavior of the inks is important, as it determines the time scale for structural recovery of the ink after flowing through the nozzle. The stresses required to overcome the yield stress and initiate flow results in structural disruption of the ink network, requiring time to recover postdeposition.^5,9,10^ The ranges of rheological values for effective DIW processing are very dependent on the polymer matrix chemistry/formulation and whether fillers are included.^11^

Without the proper viscoelastic behavior, bead spreading may occur, leading to poor shape fidelity or a failed print.^5,9,10,12^ There are two main pathways available to tune the rheological behavior of the precursor inks. The first one is varying polymer content in the ink, which research has shown that for most hydrogels/precursor gels, including chitosan (CS), higher loadings of polymer increase storage and loss moduli, as well as decreasing tan delta, indicating a more predominant solid-like behavior.^13^ Second is the addition of rheological modifiers, often a solid filler, that creates a weak three-dimensional structure in the system. This structure results in a solid-like behavior at rest that can be disrupted with shear to facilitate printing through enhanced shear thinning behavior but can reform the structure after shear is ceased.^5,14^ Some common fillers used as rheology modifiers in DIW include fumed silica, nanoclays, and graphene oxide.^5^ In addition, titanium dioxide (TiO_2_) can also be used as a rheology modifier in extrusion-based AM due to its significant influence on the resistance to flow of the material,^15^ which can result in improved bead shape retention.^11^ TiO_2_ has been shown to improve bead shape retention in DIW due to increases in yield stress and/or viscosity of the inks.^16–18^ However, most works in the DIW of TiO_2_ focus on systems with small percentages of the polymeric phase, which is used as a binder, and they do not perform a comprehensive analysis of the effect of TiO_2_ on the rheological behavior of the material. In addition, understanding the effect of different polymeric concentrations on the rheology of precursor gels/hydrogels is important for effective hydrogel printing.

To expand the current understanding of the effect of functional particles on the rheology of precursor gels/hydrogels and correlation with shape retention in DIW printing, we study a model system of CS with graphene and TiO_2_, which are functional particles commonly used for water remediation applications.^19–22^ Zetterholm et al. showed that the production of functional polymer composites of CS–graphene facilitates 90% removal of cyanotoxins and 15% removal of cyanobacteria from water after 24 h, being an effective way to deploy graphene in waterbodies by confining it in a CS matrix.^19^ Kennedy et al. reported that lattice structures of PLA with 34 wt % of TiO_2_ printed through fused filament fabrication could remove approximately 85% of cyanotoxins and 73% of algae from water over 24 h.^21^ In addition to studying the efficacy of photocatalytic TiO_2_ in degrading cyanotoxins, the authors showed that using AM to print lattice structures opened doors to more effective geometries that could boost water remediation systems.^21^

CS is an appropriate choice of hydrogel for a model system to understand the rheology of functional hydrogels in DIW, as it has been previously used in precursor inks for DIW, especially in recent years, due to its biocompatibility, biodegradability, and low cost.^23^ Examples of CS utilization in DIW include the work of Zhou et al., who published a procedure to print strong CS hydrogels based on temperature to achieve ink solidification, without the use of toxic solvents.^24^ Ajdary et al. was able to print CS combined with nanocellulose fibrils for applications in implant mesh matrices due to its noncytotoxicity.^25^ Laurén et al. utilized CS with nonisocyanate polyurethane/cellulose nanofiber for wound healing applications.^26^ Marapureddy et al. published their work on CS with graphene oxide, in which they leveraged the geometric freedom and particle alignment imparted by the DIW manufacturing to facilitate cell differentiations.^7^ However, most papers do not perform a systematic comparison of how the rheological behavior of CS inks relates to different degrees of bead spreading, much less when functional particles are included.

In our systematic study of a model CS–G–TiO_2_ system, we focus on the shape retention aspect of DIW printability and how it can be improved based on tuning the rheological behavior of the system. Understanding the isolated rheological parameters that affect bead spreading is important to optimize DIW printing of inks. We focus on understanding not only whether fillers/polymer content lead to samples with a predominant solid-like behavior, but we highlight how tan delta can be utilized to predict the time scale at which a predominant solid-like behavior will be present in each of the samples. This is especially important when processing samples in which a solidification step is only performed at the end of printing the whole part, where longer time scales are expected between printing and solidification of the material.

In this work, a CS–G–TiO_2_ system is used as a model to understand the impact of polymer and functional particle content (rheology modifiers) on the rheology and bead spreading behavior of filled hydrogel systems. Our study focuses on the effect of each individual formulation component (CS, graphene, and TiO_2_) on the rheological and viscoelastic properties of CS–G–TiO_2_ inks. We explore the effect of fillers on tan delta and on the stress at the transition from solid-like to liquid-like behavior of materials, comparing them to the bead shape retention of formulations. With an understanding of the effect of rheology on bead spreading, we print multilayer 3D parts to assess their behavior in more complex structures. This work contributes to a broader understanding of the impact of rheology modification with active functional particles on DIW AM, particularly in the area of hydrogel materials, which can lead to more efficient production of polymer-composite-based DIW hydrogel inks through rheological characterization.

## 2 Materials and Methods

### 2.1 Materials

CS–G–TiO_2_ composites were prepared using CS (190–310 kDa, deacetylation degree of 75–85%, *Millipore Sigma*), C750 graphene nanoplatelets (750 m^2^/g, *XG Sciences*), ultrapure water (18 MΩ·cm), acetic acid (glacial, *Millipore Sigma*), and P25 TiO_2_ (>90% anatase, 25 nm particle size, 50 m^2^/g, *Degussa*).

### 2.2 Ink Fabrication Methodology and Characterization

CS in its natural form is a water-insoluble polymer, which cannot be dissolved in water for the production of hydrogels.^27,28^ However, the addition of a weak acid in the CS–water mixture, commonly acetic acid, causes the amine groups in CS to partially protonate.^27,29^ This protonation step results in a water-soluble partially protonated CS (p-CS), allowing it to be dissolved in water.^29^ p-CS can be deprotonated in contact with a basic solution, commonly 1 M sodium hydroxide, returning to its water-insoluble state.^28^ The ability to be deprotonated back to the water-insoluble state allows CS to be used in water remediation applications since its water solubility can be controlled.

CS–G–TiO_2_ inks were prepared with CS ranging from 5 to 9 wt %, graphene from 0 to 2 wt %, and TiO_2_ from 0 to 25 wt %. A Box–Behnken design of experiment (DOE) was used in the statistical software package Minitab (Minitab LLC, PA, USA) to study the statistical significance of each formulation component on the properties of the ink. The DOE evaluated 15 ink formulations within the desired composition ranges, creating a response surface. Thirteen formulations were unique, and the midcompositional point (7% CS, 1% G, and 12.5% TiO_2_) was replicated three times to assess the experimental error. All compositions studied in this article are shown in Table S1. The DOE results are shown in Table S3 through Table S7 and Figure S15 through Figure S18.

The procedure followed in this paper for the production of CS–G–TiO_2_ inks for DIW is based on the work of Zetterholm et al.,^19^ for the production of CS–G films for water remediation applications. Their procedure involves preparing a solution of CS–G in water with acetic acid, which is cast to evaporate the solvents and form a CS–G film. However, the viscosity of the solution is low and inadequate for DIW printing. Therefore, we modified the procedure to prepare higher viscosity CS–G–TiO_2_ inks in water with acetic acid, allowing printing with DIW. The first modification step was decreasing the amount of water in the solution (keeping acetic acid concentration constant) to increase the concentration of solutes (CS, G, and TiO_2_). However, this modification resulted in inconsistencies and batch-to-batch variations in the rheological properties of the inks. After further investigation, we hypothesized that the lower water content in the inks, which resulted in a higher viscosity material, decreased the mobility/diffusion of water/acetic acid in the CS–G–TiO_2_ mixture. This led to significant variations in the degree of partial protonation of CS depending on the concentration of the solute (CS–G–TiO_2_). To avoid the dependency on solute concentration on the degree of partial protonation of CS, CS was protonated prior to preparing the inks in water with acetic acid. The procedure involved adding 1 wt % of CS to ultrapure water with 1.5 vol % of acetic acid and then mixed in a stirring plate at medium-high speed for 48 h at room temperature. The solution was then cast in a polypropylene mold to evaporate the solvents for 48 h to obtain a film of p-CS. p-CS films were cryogenic ground with a mortar and pestle using liquid nitrogen to obtain a powder of water-soluble p-CS.

For the production of CS–G–TiO_2_ inks, graphene and TiO_2_ were dispersed in ultrapure water using a Branson 5800 ultrasonic bath (37 kHz frequency) for 1 h at room temperature, which is the same procedure published by Zetterholm,^19^ except for the addition of TiO_2_. After dispersion, p-CS powder was added to the solution and mixed with a Vortex Mixer (USA-LAB-MX) for 10 s to homogenize the ink. Different from the work of Zetterholm et al.,^19^ no further acetic acid is required, since CS is already partially protonated at this point. The CS–G–TiO_2_ ink is rested for 48 h to allow p-CS to completely solubilize. Finally, a FlackTek mixer model DAC 1200–300 VAC was used to mix the ink for 5 min, with a mixing speed of 2000 rpm during minutes 1, 3, and 5 and a speed of 500 rpm during minutes 2 and 4, to avoid overheating the sample due to mixing friction. The aforementioned procedure resulted in consistent rheological properties of CS–G–TiO_2_ inks.

Since the required changes in the procedure from the work of Zetterholm et al.^19^ do not require acetic acid to be added after the particle ultrasonication step, a cursory analysis was performed to explore whether acetic acid is necessary during particle dispersion. A particle size analyzer was used (the methodology is discussed next in Section 2.3) to measure the size of graphene and TiO_2_ agglomerates in water, shown in Figure S1, which indicated that the presence of acetic acid led to larger graphene agglomerates and does not affect particle agglomeration for TiO_2_. These results are confirmed with visual analysis and SEM (Figures S2 and S3). Therefore, the particles were dispersed in water with no acetic acid, followed by the incorporation of p-CS to form CS–G–TiO_2_ inks.

### 2.3 Particle Size Characterization and Sample Morphology

The agglomerate sizes of graphene and TiO_2_ were characterized using a Horiba LA-950 particle size analyzer using water as the medium. The particle concentration was automatically adjusted by the instrument until the blue and red transmittance were at 80 and 55%, respectively. Samples were dispersed in the ultrasonic bath prior to particle size characterization in either ultrapure water or in ultrapure water with 1.5% acetic acid.

Sample morphology and qualitative particle distribution in the inks were studied by using Scanning electron microscopy (SEM) and energy-dispersive X-ray spectroscopy (EDS). Prior to SEM analysis, samples were freeze-dried at −50 °C overnight using a Labconco FreeZone 2.5 L Benchtop Freeze-Dryer to remove water while maintaining their morphology. Samples were placed on aluminum stubs with carbon tape and then sputtered with a 10 nm thick layer of iridium to increase conductivity. A JEOL IT-500HR equipped with EDS was used to analyze the cross-section of cryofractured samples using an accelerating voltage of 10 kV.

### 2.4 CS–G–TiO_2_ Ink Rheological Characterization

A TA Instruments HR-2 rheometer with a 25 mm stainless steel parallel plate geometry, a Peltier plate, and a solvent trap were used to measure rheological properties of the inks. Tests were conducted with a 1 mm gap at 22 °C. All samples were presheared at 0.5 s^–1^ for 10 s followed by a 60 s resting interval prior to testing.

Amplitude sweep tests were conducted from 0.01 to 500% at an angular frequency of 1 and 100 rad/s to measure the limit of the linear viscoelastic region of each sample. The upper strain of the linear viscoelastic region was defined as the strain at which a 2.5% decrease in the storage modulus was observed. The stress at the crossover point between the storage and loss modulus was also identified.

Frequency sweep tests were used to obtain the viscoelastic properties of the inks. Tests were conducted from 0.04 to 100 rad/s at the largest amplitude strain where the ink was still in the linear viscoelastic region, which varied for each sample, as shown in Table S1 in the Supporting Information. Three-interval thixotropy tests (3ITTs) were used to measure the storage modulus recovery of the inks. Test intervals 1 and 3 were low deformation intervals performed at 10 rad/s and 0.05% strain for 60 and 90 s, respectively. Test interval 2 was a high deformation interval performed at 10 rad/s and 200% strain for 20 s (unless otherwise specified). A fresh sample was utilized for each test.

### 2.5 Direct Ink Write Printing of CS–G–TiO_2_ Inks

The CS–G–TiO_2_ inks were printed on a custom-built 3-axis DIW printer having 500 mm of travel in each of the three axes. All of the ink compositions were extruded using a Nordson Ultimus V pressure dispenser and a 10 cm^3^ HPx High-Pressure Dispensing Tool capable of producing up to 400 psi of extrusion pressure. The inks are screened for extrudability using a variety of Nordson SmoothFlow tapered nozzles ranging from 0.58 to 1.6 mm. For the ink compositions that extruded successfully, a bead spreading characterization was conducted to quantify the inks’ printability. A 0.58 mm diameter tapered nozzle was used to print three beads 50 mm long on a glass slide at a translation speed of 700 mm/min. A Keyence LJ-v7000 laser profilometer was then used to measure the beads spreading over a period of 5 min at a fixed location. The bead profiles were recorded at 0, 30, 60, 120, and 300 s. To have a consistent comparison of bead spreading across different ink compositions, the bead width and height data for each individual ink composition were normalized to a maximum value of 1 mm.

For multilayer parts, samples were printed with a 1 mm height, printing speed of 600 mm/min, and pressures of 10.4 psi for CS(5)–G(1)–TiO_2_(0), CS(5)–G(1)–TiO_2_(12.5), and 33 psi for CS(7)–G(1)–TiO_2_(12.5) samples.

## 3 Results and Discussion

### 3.1 CS–G–TiO_2_ Morphology

Sample morphology and qualitative particle distribution were analyzed using SEM and EDS. In order to preserve morphological features as closely as possible, samples were freeze-dried prior to SEM analysis. Figure S4 shows the morphology of a CS(7)–G(0)–TiO_2_(0) sample that contains only CS in its formulation. The CS-only sample shows a suspected open-cell 3D network formed by CS. Some larger pores in the range of 100 μm are observed as well as smaller pores around 10 μm. The formation of a 3D network in CS inks could be beneficial for water treatment due to an increased surface area which increases the frequency of interactions between the sample and water.

CS samples with graphene and TiO_2_ were analyzed by SEM and EDS to examine particle distribution in the composites (Figure S5). SEM shows that with the addition of particles, a 3D network of CS can still be observed. In addition, particles can be seen distributed across the network. EDS elemental mapping shows a homogeneous distribution of titanium in the sample, confirming SEM results that TiO_2_ is well distributed in the samples.

### 3.2 Rheology

#### 3.2.1 Yield Stress Analysis

Rheology measurements probed the oscillatory strain amplitude response of samples. The crossover point between storage (*G*′) and loss (*G*″) moduli was analyzed, which indicates the point at which the ink changes from a predominant solid-like behavior to a predominant liquid-like behavior.^8^ Higher stresses at the crossover point represent that a higher pressure is required for the ink to begin flowing in the barrel and through the nozzle during DIW printing, which impacts printer selection and processing parameters. Figure 1 illustrates stress at the *G*′/*G*″ crossover point of all formulations.

**Figure 1:**
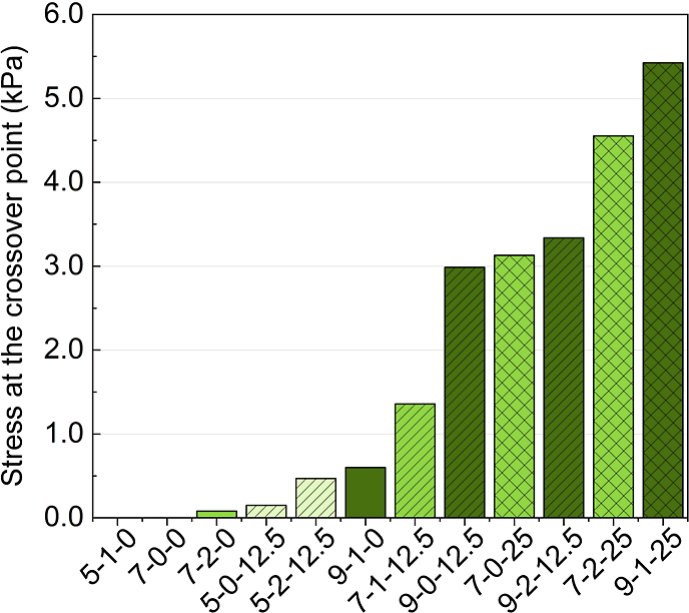
Stress required for the material to go from predominant solid-like to predominant liquid-like behavior (crossover point between *G*′ and *G*″). Increasing CS, G, or TiO_2_ content leads to increases in required stress.

By increasing the content of any of the formulation components, an increase in the stress at the crossover is observed. This increase is especially prominent for the addition of TiO_2_. Samples with a stress value of 0 kPa indicate that no crossover between *G*′ and *G*″ was observed because they displayed a predominant liquid-like behavior at all tested strains. Therefore, increasing the content of solid fillers and polymer leads to a more predominant solid-like behavior, which can aid in better bead shape retention of a printed bead. In the case of CS being added to the system, more physical interactions and more entanglements in the polymer chains are expected, limiting the flow and increasing the solid-like behavior of the material,^30^ demonstrated here through increases in the stress required for the material to flow. The same behavior is seen when adding particles to the formulations as they can create a steric hindrance to the movement of the polymer chains, which increases their resistance to flow. In the case of TiO_2_, physical interactions in the form of hydrogen bonds may also happen,^31^ further increasing polymer–particle interactions and resulting in increases in the stress required for the material to flow. However, just understanding the stress required to print a sample is not sufficient, as these materials usually display thixotropic (time dependent) behavior.

In addition to the crossover point analysis, the magnitude of the complex viscosity as a function of angular frequency of all samples was measured, shown in Figure S6, showing that compositions with higher loadings of particles display higher magnitudes of the complex viscosity, especially at low angular frequencies.

#### 3.2.2 Thixotropic Behavior of Samples from Three Interval Thixotropy Tests

During DIW printing, inks are mainly subjected to three different deformation intervals. First, as the ink sits in the barrel, it is subjected to lower strains at low shear rates. Second, when it reaches the nozzle, high-shear rates are present due to the variation in diameter from the barrel to the nozzle, resulting in high strains. Lastly, the ink is deposited to form a 3D shape, again subjected to low strain and rate. Figure 2 exemplifies the three regions.

**Figure 2:**
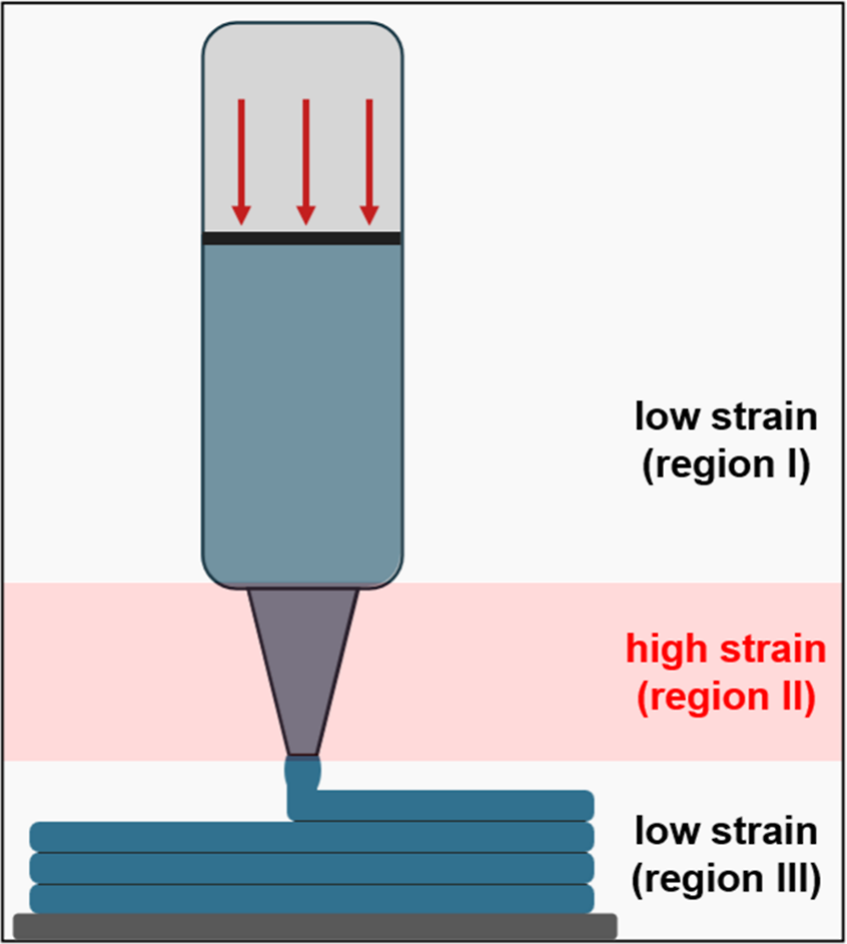
Representation of the three main strain regions in direct ink writing. Region I and region III are low-strain regions, while region II is a high-strain region due to the higher shear rates that the material is subjected to flow through the nozzle. This image visualizes the justification for implementing 3ITT to characterize rheological behavior in DIW.

Ideally, some yield stress behavior is required in region I, to avoid dripping and, especially, in region III, to facilitate shape retention. In region II, a more predominant liquid-like behavior is desired for extrudability. Depending on the formulation of the ink, the viscosity at rest of the material may be different before and after the high-strain interval. For example, when a weak particle network is formed in the material, this network can be disrupted by the high-shear rates associated with the nozzle region. The time required for the reformation of this network after shear varies depending on the matrix and particle used.^32^ Therefore, assessing the rheological behavior after a high-strain interval is crucial to understanding recovery of fundamental rheological parameters in the context of a DIW process.

An effective way to study the solid-like recovery after a high strain interval is through a three interval thixotropy test (3ITT). In this rheological test, the material is subjected to three intervals at different strains. The first and third intervals are at low strains, simulating regions I and III of the printing process. The second interval is a high-strain interval representing the region where the material flows (region II). This test allows for a comparison of variation in *G*′ and *G*″ due to high strains. Results of 3ITT for some representative samples are shown in Figure 3. G', G'' and 3ITT results for all samples are shown in Figures S7 and S8 of the Supporting Information.

**Figure 3:**
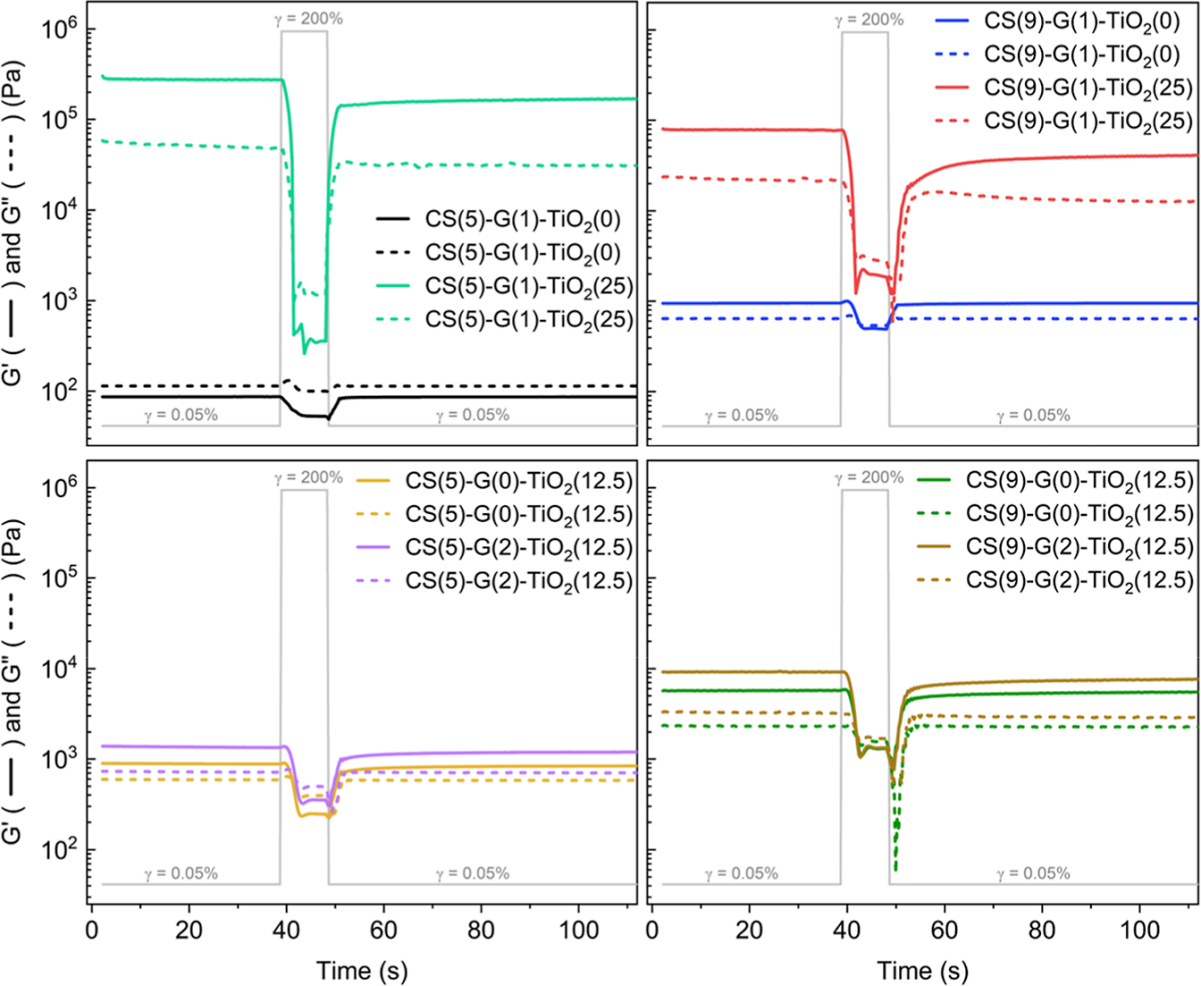
3ITT of representative CS–G–TiO_2_ formulations. Intervals 1 and 3 are low deformation intervals of 0.05% strain, and interval 2 is a high deformation interval of 200% strain. Different recoveries after high-strain intervals are observed based on the ink formulation (data was treated with 7 points AAv smoothing).

Interval 1 of the 3ITT was utilized to gather baseline properties. Interval 2 enabled sufficient disruption of the interparticle network, displaying a predominant viscous-like behavior (*G*″ > *G*′). Interval 3 is identical to interval 1 to assess the recovery of the material as a function of time. Data in Figure 3 was smoothed using a 7 points AAv smooth, which clustered data points into averages of 7 points to reduce noise, which is present especially due to the abrupt change of strain between intervals.

The percentage of recovery of *G*′ after interval 2 (high-strain interval) is analyzed as a function of time to understand how *G*′ is affected by periods of high strain. Recovery was calculated using eq 1.

1 $$G_{r}^{\prime }=\frac{G_{3}^{\prime }}{G_{1}^{\prime }}$$

where *G*′_r_ is the recovered storage modulus, *G*′_3_ is the storage modulus after interval 2, and *G*′_1_ is the storage modulus before interval 2. Values of *G*′_r_ that reach 1 in the time scale tested indicate full recovery of the storage modulus in this time scale (*G*′_3_ = *G*′_1_), indicating that the material could reform after the high-strain interval back to its original behavior. Recovery values smaller than 1 indicate that the storage modulus is smaller after the high-strain interval, indicating structural changes in the material. Figure 4 displays the recovery for some of the representative formulations. Results for all curves can be seen in Figure S10 in the Supporting Information.

**Figure 4:**
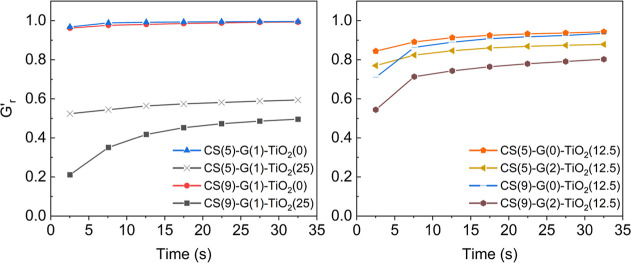
*G*′ recovery after high deformation interval in 3ITTs. The result at each time point is obtained from the average of the 5 s around it (e.g., the 2.5 s data point is the average of interval from *t* = 0 to *t* = 5 s).

Samples prepared without TiO_2_ showed an almost instantaneous recovery in tan delta, reaching a recovery value of 1 in a few seconds. Meanwhile, formulations with higher loadings of particles show lower recoveries after high strains, indicating a disruption of particle interactions during higher strains. The combination of higher concentrations of CS (9 wt %) with 25 wt % of TiO_2_ shows a smaller recovery than a smaller concentration of CS (5 wt %) with 25 wt % of TiO_2_. This effect is also seen when graphene is combined with intermediate values of TiO_2_ (12.5%). However, when no TiO_2_ or smaller amounts (12.5 wt %) of TiO_2_ are present, without the presence of graphene, increasing the concentration of CS does not play a significant role in decreasing the ability of the formulation to recover after a high-strain interval. Therefore, it is shown that the effect of CS on the recovery of the inks is not as detrimental as the addition of particles, likely because interactions between chains are not as easily disrupted and can reform more easily.

#### 3.2.3 Tan Delta and Its Behavior in the Time Scale of AM Processing

Tan delta is the ratio of loss modulus (energy dissipated in the system) to storage modulus (energy stored in the system). Tan delta values higher than 1 indicate a predominant viscous response (energy dissipation), while tan delta values smaller than 1 represent materials with a predominant elastic response (energy storage).^8^ Tan delta as a function of angular frequency probes the viscoelastic response of the material at different time scales as angular frequency is inversely related to time. At lower angular frequencies, the behavior is characteristic of longer times, and at higher angular frequencies, the behavior indicates a shorter, more instantaneous response.^8^ Tan delta values decrease as a function of angular frequency, which is the common behavior observed in viscoelastic materials. With less time to rearrange and dissipate energy, the response of the material will be more elastic, therefore showing lower tan delta values. Most samples displayed tan delta values below 1 for the range of angular frequencies studied, indicating a dominant elastic response of the formulations. A dominant elastic response is desired for structural stability of the material after printing, especially at low angular frequencies, indicating structural stability at longer time scales.^8^

Tan delta values were measured as a function of angular frequency and are shown in Figure 5 for select formulations. Tan delta values as a function of angular frequency of all samples are shown in Figure S11 as well as tan delta values during 3ITTs (Figure S12).

**Figure 5:**
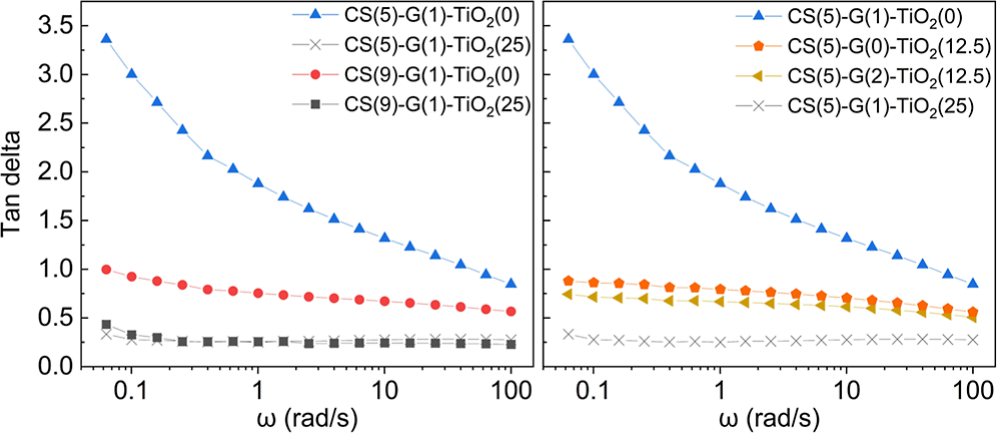
Tan delta as a function of angular frequency for some representative DOE formulations. Increasing CS content or adding particles to the inks leads to a lower tan delta response.

Samples with lower CS and TiO_2_ contents displayed a more viscous response (tan delta >1), not ideal for the bead to retain its shape. Increasing CS content translates into a more elastic response, as more chain interactions are occurring, forming a stronger network in the ink. The addition of particles also contributes to a more elastic response, likely by hindering the rearrangement of the material’s structure under stress, and, therefore, the dissipation of energy. From the comparison of formulations shown in Figure 5, TiO_2_ concentration was the factor influencing tan delta the most. This was confirmed by the design of experiment analysis of tan delta shown in Figure S17.

Even though the tan delta values as a function of angular frequency for the ranges tested in Figure 5 give a good indication of the solid-to-liquid-like ratio of samples, it is not sufficient to understand the behavior of materials at rest. For example, a CS(9)–G(1)–TiO_2_(0) sample shows values of tan delta smaller than or equal to 1 for the whole range of angular frequencies tested, but it shows a trend that at even lower angular frequencies (longer time scales), its behavior would be more viscous dominated (tan delta >1). This is also observed from 3ITT results (Figure 3), in which an apparent predominant solid-like behavior is observed after the high-strain interval for most samples. However, these results do not necessarily describe the entirety of the sample behavior during processing, because it does not consider longer time scales. For example, when printing multilayer parts (Figure 10), in which a solidification step would only be performed at the end of printing the whole part (at which a “true” yield stress behavior would be obtained), a predominant solid-like behavior is required for the extent of the entire printing process (dependent on printing speed and part size). Therefore, to compare the tan delta behavior of different formulations at different time scales, a mathematical power law equation fitting (*y* = *b* × *x* ^*a*^) was performed. The values of *b*, *a*, and *R* ^2^ for all samples are shown in Table S2. From the coefficients of the power law equation fitting, the angular frequency (ω) at which a transition from solid- to liquid-like behavior (tan delta = 1) can be estimated for each sample. The angular frequency at tan delta = 1 can be used to obtain the period (*T*) associated with this transition using eq 2, giving an idea of the time scale at which each sample stays in a predominant solid-like behavior, which is desired for better shape retention.

2 $$T=\frac{2\pi }{\omega }$$

The estimated period required for samples to go from solid- to liquid-like behavior is shown in Figure 6.

**Figure 6:**
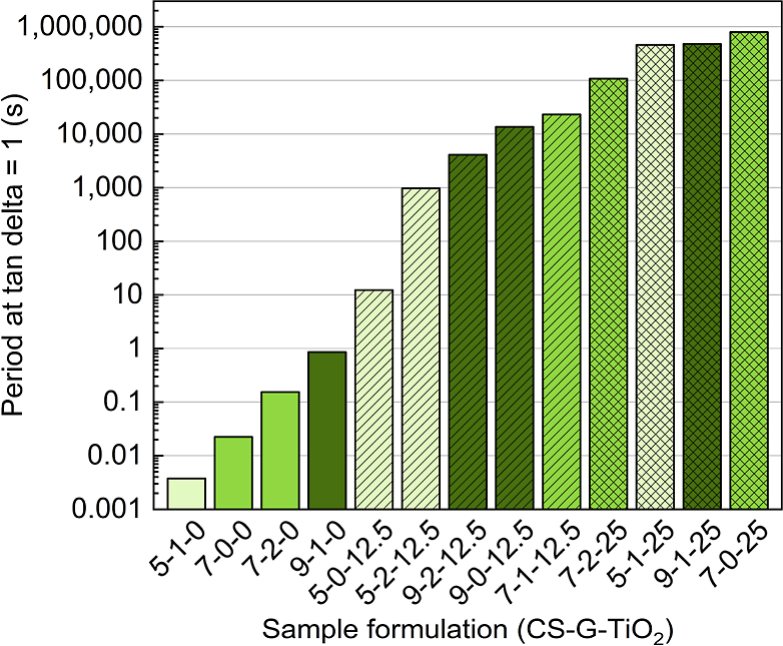
Period estimated for formulations to change from a predominant solid-like to liquid-like behavior (tan delta = 1 or *G*′ = *G*″).

The estimated period required for the solid- to liquid-like transition (tan delta = 1) is mostly dependent on TiO_2_ content, as also seen in the absolute tan delta values (Figure 5). With a higher loading of TiO_2_, a more solid-like response is observed. At lower TiO_2_ content, the impact of CS and G is also observed, with both increasing the period required for the material to change to a predominant liquid-like behavior and increasing the solid-like response. Longer solid-like times could favor bead shape retention at longer time scales, which is required for printing multilayer parts.

### 3.3 DIW Printing Analysis: Tan Delta as an Effective Bead Spreading Indicator

Inks containing no TiO_2_ showed predicted extrusion pressures in the range of 5 to 22 psi (volumetric flow rate of 4 mL/min and nozzle size of 0.58 mm), with 5 wt % CS ink having the lowest extrusion pressure and 9 wt % CS ink having the highest extrusion pressure. In line with the rheology results (from the stress required to go from solid-like to liquid-like behavior), the predicted extrusion pressure increased with an increase in TiO_2_ content. Samples containing 12.5 wt % TiO_2_ showed a predicted pressure range of 18–95 psi for a nozzle size of 0.58 mm and a volumetric flow rate of 4 mL/min. For the same nozzle size and flow rate, the CS(5)–G(1)–TiO_2_(25) sample showed a predicted pressure of 288 psi and the CS(9)–G(1)–TiO_2_(25) sample showed a predicted pressure of 710 psi, which was higher than the maximum pressure the extruder can produce (400 psi). For the CS(9)–G(1)–TiO_2_(25) sample, the predicted extrusion pressure (411 psi) exceeded the maximum pressure even after using the largest available nozzle size of 3 mm. However, experimentally, the ink was able to extrude at a maximum pressure of 400 psi, even though the extrusion was highly inconsistent and can be considered not extrudable for uniform DIW printing.

Similarly, during experimental testing of the extrusion of CS(5)–G(1)–TiO_2_(25) and CS(7)–G(0)–TiO_2_(25) inks, extrusion was not observed with a nozzle size of 0.58 mm. Upon using a 3 mm nozzle, these inks extruded but displayed highly inconsistent extrusion. There was no direct relationship between stress at the crossover point and whether or not a certain ink could be extruded. All the inks that could not be extruded properly were the ones with 25 wt % of TiO_2_, indicating that a higher concentration of these fillers can lead to inconsistent extrusion. For example, the stress at the crossover point of CS(9)–G(1)–TiO_2_(12.5) is similar to that of a CS(7)–G(0)–TiO_2_(25) sample, but the sample with less TiO_2_ could be extruded, while the sample with more TiO_2_ could not (Figures 7 and 8). As a result, all the inks containing 25 wt % TiO_2_ were considered to be not extrudable. Figure 8 illustrates the printed roads of inks containing 25 wt % of TiO_2_.

**Figure 7:**
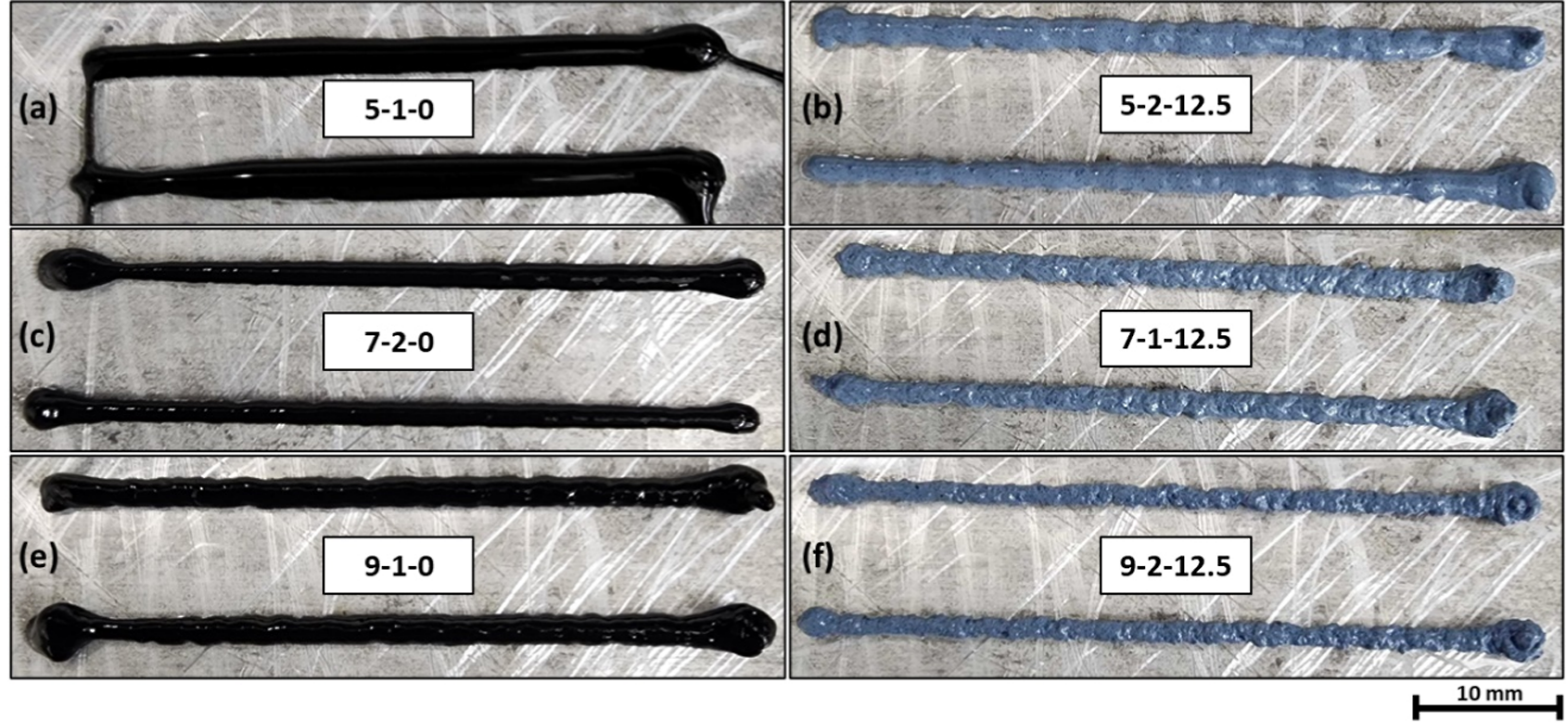
Visual representation of printed single-layer beads of CS–G–TiO_2_ formulations after 300 s (road length of 50 mm). Low particle contents resulted in poor shape retention (5–1–0). Intermediate compositions with 12.5 wt % of TiO_2_ showed good shape retention and extrudability. (a) CS(5)–G(1)–TiO_2_(0); (b) CS(5)–G(2)–TiO_2_(12.5); (c) CS(7)–G(2)–TiO_2_(0); (d) CS(7)–G(1)–TiO_2_(12.5); (e) CS(9)–G(1)–TiO_2_(0); (f) CS(9)–G(2)–TiO_2_(12.5).

**Figure 8:**
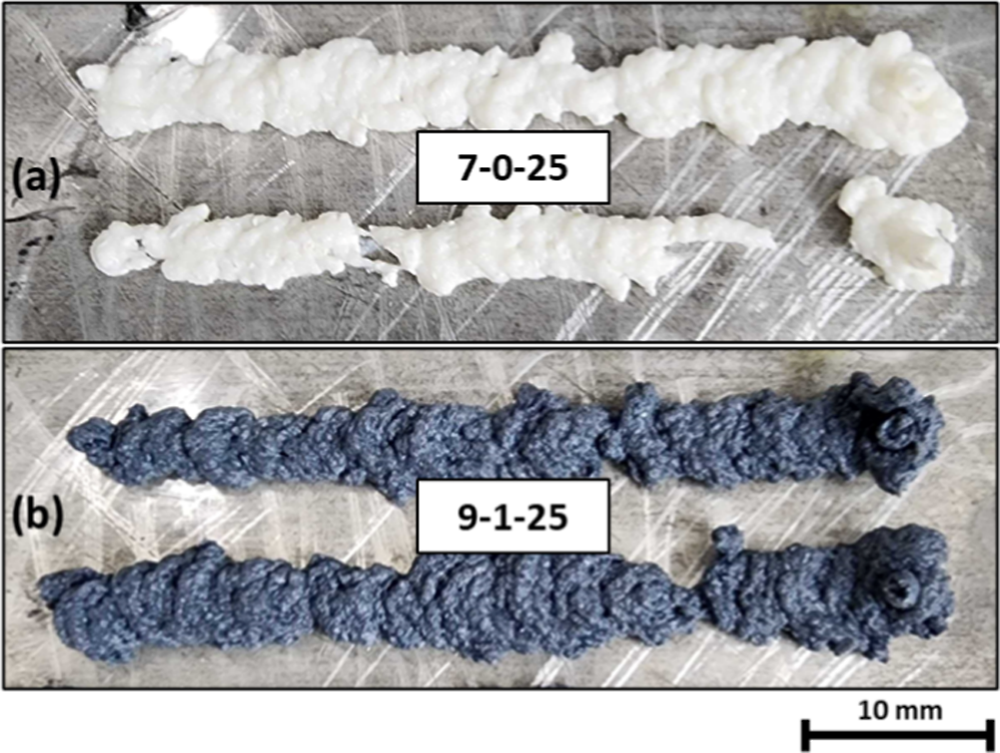
Visual representation of printed single-layer beads of CS–G–TiO_2_ formulations after 300 s (road length of 50 mm). High particle content led to inconsistent extrusion and printing defects. (a) CS(7)–G(0)–TiO_2_(25); (b) CS(9)–G(1)–TiO_2_(25).

For the inks that showed uniform extrusion, the bead width was monitored for spreading as a function of time to compare the printability of the deposited beads to their rheological behavior. This was done to quantify the behavior of the inks at AM-relevant length scales considering a layer time of around 15–30 s and time taken to build a multilayer part of around ten layers (300 s). One of the limitations of using a laser profilometer to measure the bead spreading was that it could not accurately measure transparent (e.g., CS(7)–G(0)–TiO_2_(0)) or white samples (samples without graphene in them), which limited a thorough comparison of all DOE formulations. Figure 7 presents the visual aspects of printed samples.

Visual and profilometry analyses (Figure 9) of printed beads help identify some trends with respect to the formulations. Due to the relatively minor influence of graphene on the properties, the discussion focuses on the impacts of varying CS and TiO_2_ concentration. First, a visual analysis of the CS(5)–G(1)–TiO_2_(0) formulation (Figure 7(a)) and bead width as a function of time (Figure 9) shows that the ink starts spreading right after deposition and continues spreading over the period of 5 min. Spreading of the deposited beads leads to poor shape fidelity, as seen in Figure 7, and will tend to get worse as subsequent layers are deposited. This behavior of the CS(5)–G(1)–TiO_2_(0) ink was expected from tan delta results, which indicated predominantly viscous behavior at low angular frequencies (tan delta >1 at low angular frequencies), indicative of no structural stability at longer time scales. This is also observed in the 3ITT curve where the CS(5)–G(1)–TiO_2_(0) ink behaves liquid-like (tan delta >1) throughout all three strain regions, indicating that the ink will continue to spread after deposition, as no predominant structural formation is observed.

**Figure 9:**
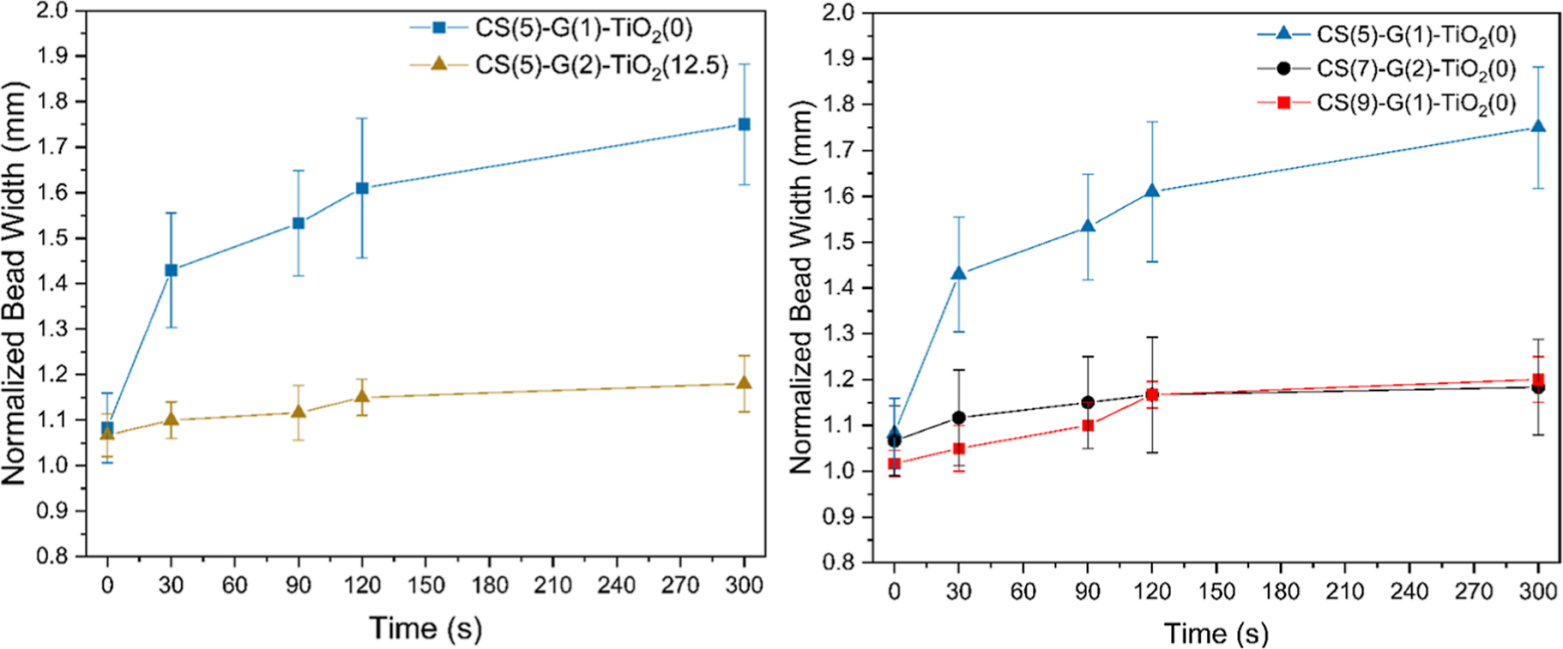
Bead width variations as a function of time for samples. (Left) Keeping CS content at 5 wt % and varying TiO_2_. (Right) Varying CS from 5 wt % to 9 wt % while keeping TiO_2_ content constant. Addition of particles and/or increasing CS content leads to better shape retention.

When 12.5 wt % of TiO_2_ is added (Figures 7(b) and 9, Left), better shape retention is observed, as the addition of TiO_2_ changes the viscoelastic behavior to predominantly elastic (tan delta <1 at low angular frequencies). The CS(5)–G(1)–TiO_2_(12.5) ink does not spread appreciably after deposition and continues to hold its as-deposited shape even after 5 min. The minimal variation observed in Figure 9 is due to measurement noise, and the difference between the widths at different time intervals is statistically insignificant. This measurement is also in accordance with the 3ITT where the ink displays predominantly elastic behavior (tan delta <1) after being relieved from high strain which restricts spreading (Figure S12). In addition, a smooth surface can be observed in the sample, indicative of uniform extrusion.

When increasing CS content from 5 to 7 and 9 wt % (Figure 7(a) to (c) to (e) and Figure 7(b) to (d) to (f)), a less smooth surface is observed, with more variation in diameter observed across the same printed bead, which may be indicating an onset of inconsistent extrusion during printing. The bead width variation (Figure 9, Right) of CS(7)–G(2)–TiO_2_(0) and CS(9)–G(1)–TiO_2_(0) inks shows very minimal spreading compared to the CS(5)–G(1)–TiO_2_(0) ink, as an increase in CS content from 5 to 7 and 9 wt % resulted in the inks behaving predominantly “solid-like” (tan delta <1) under low strains. The CS(9)–G(2)–TiO_2_(12.5) sample (Figure 7(f)) shows more inconsistencies than CS(5)–G(2)–TiO_2_(12.5) and a similar behavior to that of CS(7)–G(1)–TiO_2_(12.5) but with slightly more surface defects. Inks with 9 wt % could still easily be printed. On the other hand, by increasing TiO_2_ content to 25 wt %, poor extrudability was obtained, as seen in Figure 8.

Increasing TiO_2_ to 25 wt % leads to highly inconsistent extrusion resulting in a disordered bead shape, with considerate loss of shape and the presence of defects in the form of over/under extrusion. The ink was considered to not be extrudable for DIW. The extrusion behavior of inks with 25 wt % of TiO_2_ is likely linked to bigger and more frequent TiO_2_ agglomerates, which leads to inconsistent extrusion and poor extrudability. SEM images of the cross section of these printed beads are shown in Figures S13 and S14. The normalized bead widths of some samples that could be adequately printed are shown in Figure 9.

Visual and profilometry analysis indicates 3 clear behaviors for CS–G–TiO_2_ formulations when printed. First, samples with a viscous-dominant viscoelastic response (tan delta >1 at ω = 0.06 rad/s) tend to spread over time, not retaining the printed bead shape. For comparison, Marapureddy et al.^7^ reported tan delta values for 3 wt % CS with 0.3 to 0.5 wt % of graphene oxide samples that printed well between 0.175 and 0.25. Gao et al.^6^ reported tan delta values for alginate-gelatin samples printed well when between 0.25 and 0.45. The other two observed behaviors in this work were samples with predominant elastic behavior (tan delta <1), which tend to print well depending on the amount of TiO_2_ present. If the amount is low to medium (up to 12.5 wt %), beads would be able to retain their shape well, especially samples with a higher CS content, which displayed lower tan deltas and good structural recovery. On the other hand, samples with high solid content (25 wt % of TiO_2_) resulted in unstable printing with inconsistent extrusion and poor shape fidelity and can be generally considered not extrudable. Therefore, spreading in single beads could be predicted based on the value of tan delta of samples at low angular frequencies, with tan delta <1 showing low width variation. In our work, we generalize that even though some ranges of tan delta are optimal for adequate printability, as mentioned in the works of Marapureddy and Gao, an initial assessment of whether the formulation displays tan delta <1 can be a strong indication of its ability to showcase adequate bead spreading behavior.

To confirm that the tan delta observations can help beyond printing single layers, multilayer parts were printed by using formulations with different rheological behaviors. It is important to note that a solidification mechanism (usually through CS deprotonation with a basic solution) is required for the application of these samples in water remediation applications since the protonated ink is water-soluble. However, understanding the shape retention of the ink prior to deprotonation can facilitate printing in two main ways. First, in the case where solidification is performed after each individual layer, better shape retention of single layers can lead to better shape retention of the whole part since there is a time requirement between depositing the material and solidifying it. Second, in the case where solidification is performed after printing the whole part (usually by submerging the protonated printed sample in a NaOH bath), understanding the ability of the material to form multilayer parts without intermediate solidification steps is key for adequate printing. The behavior of inks during multilayer part printing is shown in Figure 10.

**Figure 10:**
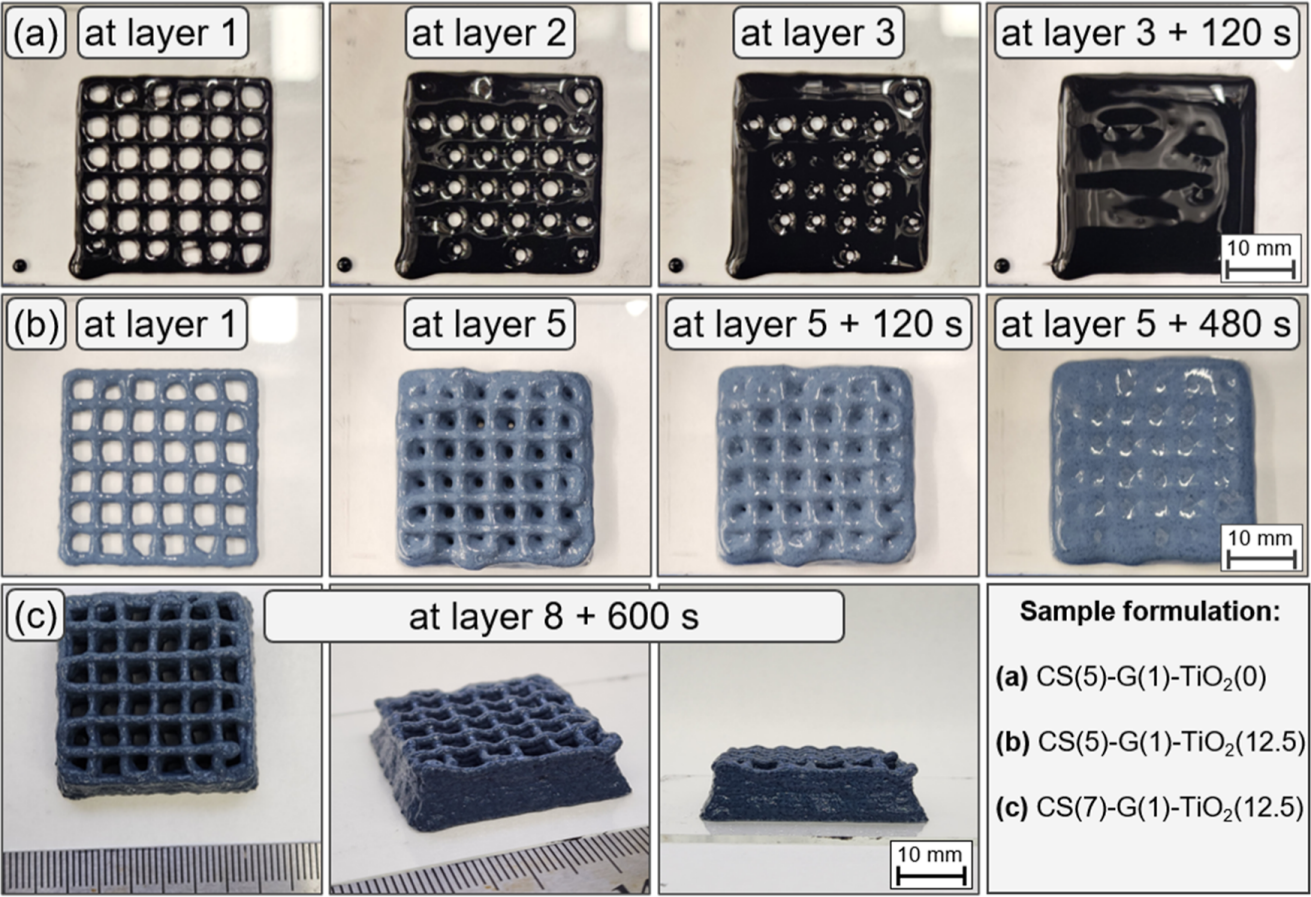
Multilayer parts printed with a layer height of 1 mm using different formulations. (Row a) CS(5)–G(1)–TiO_2_(0) samples displayed rapid slumping and could not successfully print multilayer parts. (Row b) CS(5)–G(1)–TiO_2_(12.5) samples could form multilayer parts, but it slumps in the time frame of minutes. (Row c) CS(7)–G(1)–TiO_2_(12.5) samples print well and can retain their shape, showing no perceptible visual changes in a time frame of minutes. “At layer 1” means immediately following printing layer 1. All images in row c are at the same time stamp (600 s after completing layer 8). No solidification mechanism was used during printing of these samples.

By analyzing the behavior of samples during printing of multilayer parts without the use of a solidification mechanism, we confirm the role of tan delta as a key rheological indicator for adequate shape retention in the DIW of functional hydrogels. CS(5)–G(1)–TiO_2_(0) samples, which show tan delta values at low angular frequencies larger than 1 (and an estimated period to change from solid- to liquid-like behavior of 0.004 s), could not be used to print multilayer parts due to spreading of the deposited beads. Even at layer 1, the ink could not retain its shape, showing rapid bead spreading. When 12.5% of TiO_2_ is added (sample CS(5)–G(1)–TiO_2_(12.5)), a more solid-like behavior is obtained, allowing printing of multilayer parts. The CS(5)–G(1)–TiO_2_(12.5) shows tan delta values lower than 1 at low angular frequencies, which justifies less bead spreading (Figure 9). However, tan delta values for this formulation estimated a transition to a predominant liquid-like behavior at periods in the range of hundreds of seconds. This result agrees with the visual observations that multilayer parts start to spread and lose shape fidelity at periods of time in the hundreds of seconds. Lastly, by increasing CS content to 7% (sample CS(7)–G(1)–TiO_2_(12.5)), even more solid-like behavior is seen, with tan delta values at low angular frequencies smaller than 1, and the time required for a transition from solid-like to liquid-like behavior in the thousands of seconds, agreeing with the longer shape stability of the printed samples, which displays some buckling, but no visible spreading in the minutes after printing.

Summarizing the relationship of rheology with bead shape retention (and shape retention in multilayer parts), we have shown that tan delta is a simple, yet powerful indicator of the ability of hydrogels to retain its shape after DIW printing. Samples with tan delta values larger than 1 at low angular frequencies lead to bead spreading, even in single-layer printing. For samples with tan delta values smaller than 1 at low angular frequencies, shape retention is favored in single-layer prints. For multilayer parts, the time response is also important, which shows the time scale at which the material transitions from a solid- to liquid-like response. In all of these cases, the thixotropic response of the material also defines its ability to retain its shape, measured by how long it takes for the material to return to a predominant solid-like response after high strains. No clear correlation between the stress required for the material to go from a predominant solid- to liquid-like behavior to inconsistent extrusion was observed.

## 4 Conclusions

CS hydrogels filled with graphene and TiO_2_ were studied to understand the effect of functional fillers on their rheological properties and how these properties influence extrudability and bead shape retention in DIW AM. Results showed that tan delta is the key rheological parameter for good shape retention of printed beads, with samples displaying tan delta ≤ 1 effectively maintaining their bead shape. Lower tan delta values were achieved by increasing the CS or filler content in the hydrogel. However, filler content had a greater impact on the thixotropic behavior of hydrogels than CS content, leading to a slower recovery of the storage modulus after high strains (as observed in DIW AM). Additionally, the highest filler content studied (25 wt % TiO_2_) exhibited inconsistent extrusion that hindered proper printing, a limitation not observed with increased CS content. The stress required for the material to flow increased with increasing CS, graphene, or TiO_2_ content, but it did not correlate with inconsistent extrusion, which was dependent on filler content. Multilayer parts were printed without any solidification mechanism and the results compared to their tan delta values and the time period required for the formulation to change from a predominant solid-like to liquid-like behavior. This analysis of the time-dependent structural stability obtained from tan delta values correlates well with the structural stability observations made during printing of multilayer parts. This work demonstrates that tan delta is a critical indicator for bead shape retention and spreading in DIW of hydrogels containing functional particles. Furthermore, this shows that CS–G–TiO_2_ functional inks can be tailored and successfully printed using DIW AM. The rheological insights presented here can be extended to facilitate the more efficient development of polymer composite-based hydrogel inks.

## Supplementary Material



## Notes

Additional experimental data, photography of samples, sample list, and statistical analysis (PDF)

## References

[1] Saadi M. A. S. R., Maguire A., Pottackal N. T., Thakur M. S. H., Ikram M. M., Hart A. J., Ajayan P. M., Rahman M. M. (2022). Direct Ink Writing:
A 3D Printing Technology for Diverse Materials. Adv. Mater..

[2] House A., Kuna A., Hastings D., Rodriguez N., Schoenitz M., Dreizin E. L., Guvendiren M. (2023). Effect of
particle shape on rheology and printability of highly filled reactive
inks for direct ink writing. Prog. Addit. Manuf..

[3] Nan B., Galindo-Rosales F. J., Ferreira J. M. F. (2020). 3D printing vertically: Direct ink
writing free-standing pillar arrays. Mater.
Today.

[4] Marnot A., Koube K., Jang S., Thadhani N., Kacher J., Brettmann B. (2023). Material extrusion
additive manufacturing of high particle
loaded suspensions: a review of materials, processes and challenges. Virtual Phys. Prototyp..

[5] Rau D. A., Williams C. B., Bortner M. J. (2023). Rheology and printability:
A survey
of critical relationships for direct ink write materials design. Prog. Mater. Sci..

[6] Gao T., Gillispie G. J., Copus J. S., Pr A. K., Seol Y.-J., Atala A., Yoo J. J., Lee S. J. (2018). Optimization of
gelatin–alginate composite bioink printability using rheological
parameters: a systematic approach. Biofabrication.

[7] Marapureddy S. G., Hivare P., Sharma A., Chakraborty J., Ghosh S., Gupta S., Thareja P. (2021). Rheology and direct
write printing of chitosan—graphene oxide nanocomposite hydrogels
for differentiation of neuroblastoma cells. Carbohydr. Polym..

[8] Mezger, T. The Rheology Handbook; Vincentz Network, 2020.

[9] Barrulas R.
V., Corvo M. C. (2023). Rheology
in Product Development: An Insight into 3D
Printing of Hydrogels and Aerogels. Gels.

[10] Jiang F., Zhou M., Drummer D. (2022). Effects of Fumed Silica
on Thixotropic
Behavior and Processing Window by UV-Assisted Direct Ink Writing. Polymers.

[11] Rau D. A., Bortner M. J., Williams C. B. (2023). A rheology
roadmap for evaluating
the printability of material extrusion inks. Addit. Manuf..

[12] Azimi
Yancheshme A., Yoon H., Palmese G. R., Alvarez N. J. (2024). A shape-predictive
model for the spreading of photo-curable polymers in material extrusion
additive manufacturing. Addit. Manuf..

[13] Ahmed J., Mulla M., Maniruzzaman M. (2020). Rheological
and Dielectric Behavior
of 3D-Printable Chitosan/Graphene Oxide Hydrogels. ACS Biomater. Sci. Eng..

[14] Agrawal R., García-Tuñón E. (2024). Interplay
between yielding, “recovery”,
and strength of yield stress fluids for direct ink writing: new insights
from oscillatory rheology. Soft Matter.

[15] Minagawa N., White J. L. (1976). The influence of
titanium dioxide on the rheological
and extrusion properties of polymer melts. J.
Appl. Polym. Sci..

[16] Dolganov A., Bishop M. T., Chen G. Z., Hu D. (2021). Rheological
study and
printability investigation of titania inks for Direct Ink Writing
process. Ceram. Int..

[17] Pal P., Majumder D., Rupa P. K. P. (2025). Effect
of Solid Loading of TiO2 on
The Rheological Behaviour and Microstructure of Methyl Cellulose-TiO2
Inks by Direct Ink Writing. Trans. Indian Inst.
Met..

[18] Pulido-Victoria L. A., Flores-Tlacuahuac A., Panales-Pérez A., Lara-Ceniceros T. E., Ávila-López M. A., Bonilla-Cruz J. (2025). Prediction
of viscoelastic and printability properties on binder-free TiO2-based
ceramic pastes by DIW through a machine learning approach. Comput. Chem. Eng..

[19] Zetterholm S. G., Gurtowski L., Roberts J. L., McLeod S., Fernando B. M., Griggs C. S. (2022). Graphene-Mediated
removal of Microcystin-LR in chitosan/graphene
composites for treatment of harmful algal blooms. Chemosphere.

[20] Hu X., Hu X., Tang C., Wen S., Wu X., Long J., Yang X., Wang H., Zhou L. (2017). Mechanisms underlying
degradation pathways of microcystin-LR with doped TiO2 photocatalysis. Chem. Eng. J..

[21] Kennedy A. J., McQueen A. D., Ballentine M. L., May L. R., Fernando B. M., Das A., Klaus K. L., Williams C. B., Bortner M. J. (2023). Degradation of microcystin
algal toxin by 3D printable polymer immobilized photocatalytic TiO2. Chem. Eng. J..

[22] McQueen A. D., Tedrow O. N., Ballentine M. L., Kennedy A. J. (2022). Demonstration of
Photocatalytic Degradation of Per- and Polyfluoroalkyl Substances
(PFAS) in Landfill Leachate Using 3D Printed TiO2 Composite Tiles. Water, Air, Soil Pollut..

[23] Aranaz I., Alcántara A. R., Civera M. C., Arias C., Elorza B., Heras Caballero A., Acosta N. (2021). Chitosan: An Overview
of Its Properties
and Applications. Polymers.

[24] Zhou L., Ramezani H., Sun M., Xie M., Nie J., Lv S., Cai J., Fu J., He Y. (2020). 3D printing of high-strength
chitosan hydrogel scaffolds without any organic solvents. Biomater. Sci..

[25] Ajdary R., Reyes G., Kuula J., Raussi-Lehto E., Mikkola T. S., Kankuri E., Rojas O. J. (2022). Direct Ink Writing
of Biocompatible Nanocellulose and Chitosan Hydrogels for Implant
Mesh Matrices. ACS Polym. Au.

[26] Laurén I., Farzan A., Teotia A., Lindfors N. C., Seppälä J. (2024). Direct ink
writing of biocompatible chitosan/non-isocyanate polyurethane/cellulose
nanofiber hydrogels for wound-healing applications. Int. J. Biol. Macromol..

[27] Gopi, S. ; Thomas, S. ; Pius, A. Handbook of Chitin and Chitosan; Elsevier, 2020.

[28] Wu Q., Therriault D., Heuzey M. C. (2018). Processing and Properties of Chitosan
Inks for 3D Printing of Hydrogel Microstructures. ACS Biomater. Sci. Eng..

[29] Rinaudo M., Pavlov G., Desbrières J. (1999). Influence
of acetic acid concentration
on the solubilization of chitosan. Polymer.

[30] Stojkov G., Niyazov Z., Picchioni F., Bose R. K. (2021). Relationship between
Structure and Rheology of Hydrogels for Various Applications. Gels.

[31] Siripatrawan U., Kaewklin P. (2018). Fabrication and characterization
of chitosan-titanium
dioxide nanocomposite film as ethylene scavenging and antimicrobial
active food packaging. Food Hydrocolloids.

[32] Snabre P., Mills P. I. (1996). Rheology of Weakly
Flocculated Suspensions of Rigid
Particles. J. Phys. III.

