# Supplementary material for: Rheology Indicators for Assessing Bead Spreading of Hydrogels with Functional Rheology Modifiers for Direct Ink Writing: A Case Study for Chitosan–Graphene–Titanium Dioxide
Source: ACS Appl Polym Mater. 2025 Sep 8;7(18):12846–56. doi: 10.1021/acsapm.5c02887 (PMC12481477; doi:10.1021/acsapm.5c02887)
Supplement: Supplementary file 1 [file ap5c02887_si_001.pdf]

SUPPORTING INFORMATION

Rheology Indicators for Assessing Bead Spreading of Hydrogels with Functional Rheology Modifiers for Direct Ink Write: A Case Study for Chitosan-Graphene-Titanium Dioxide

Daniel Alves Heinze [a, b, c], Supreet Thale [a,c], Yimin Yao [a, b], John P. Reynolds [d], Mark L. Ballentine [e], Christopher S. Griggs [e], Christopher B. Williams [a,c], Michael J. Bortner [a,b]*

[a] Macromolecules Innovation Institute, Virginia Tech, 240 W Campus Dr, Blacksburg, VA 24061. United States.

[b] Department of Chemical Engineering, Virginia Tech, 635 Prices Fork Rd Suite 245, Blacksburg, VA 24061. United States.

[c] Department of Mechanical Engineering, Virginia Tech, 445 Goodwin Hall, 635 Prices Fork Road - MC 0238. Blacksburg, VA 24061. United States.

[d] US Army DEVCOM Army Research Laboratory. 2800 Powder Mill Road, Adelphi, Maryland 20783-1197. United States.

[e] Environmental Engineering, U.S. Army Engineer Research and Development Center - ERDC. 3909 Halls Ferry Rd, Vicksburg, MS 39180. United States.

* Email: mbortner@vt.edu

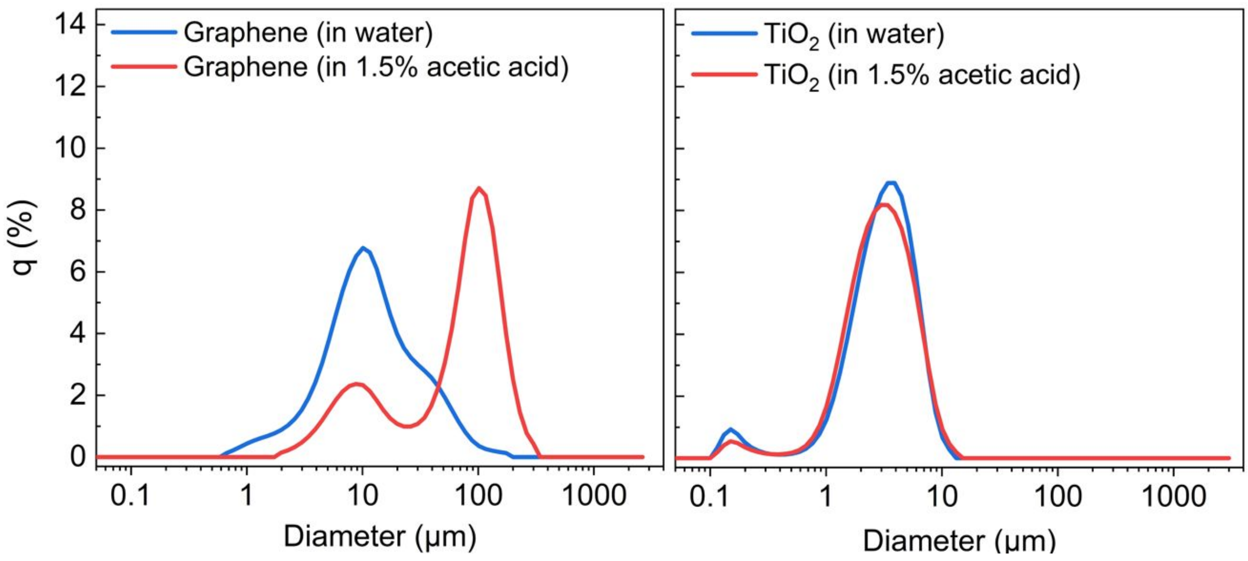

Figure S1. Particle size analysis of graphene (left) and TiO_2 (right) after 1h bath sonication in water or water with 1.5% of acetic acid. Both graphene and TiO_2 show the presence of agglomerates in the micron range, but the presence of acetic acid during sonication leads to the formation of larger agglomerates in graphene.

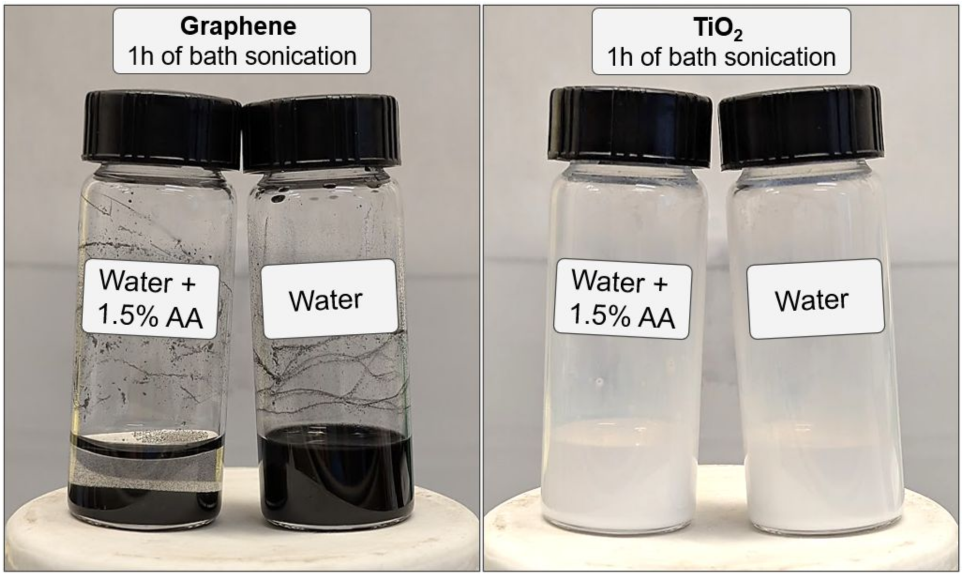

Figure S2. Visual differences of graphene and TiO_2 dispersed for 1h using bath sonication either in water with 1.5% of acetic acid (AA) or just water. Graphene in water with AA settles at the bottom of the flask, while no settling is observed in graphene sonicated in just water. No visual differences are observed for TiO_2.

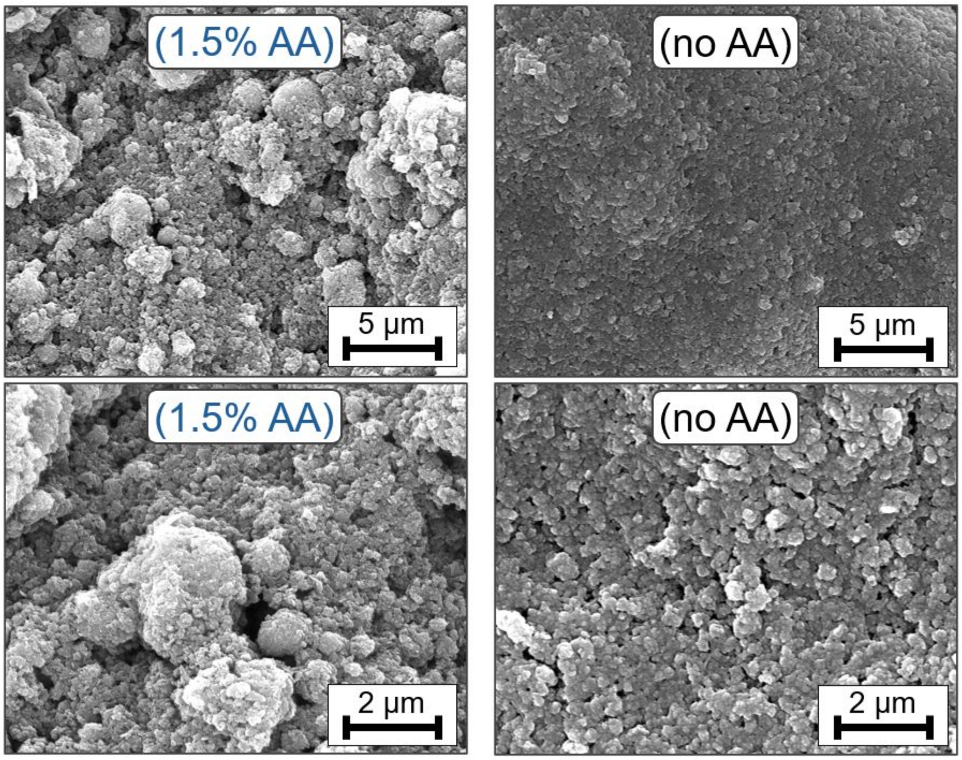

Figure S3. SEM of graphite dispersed in water with 1.5% AA (left) or just water (right). Bigger agglomerates are observed when sonicating graphene in water with 1.5% AA, agreeing with visual observations of settling and with the particle size analysis data.

Table S1. Sample compositions defined by the design of experiment and limit of the linear viscoelastic region (amplitude at which a 2.5% variation in storage modulus was observed. Smallest value chosen from tests at 1 or 100 rad/s).

**Sample composition (CS-G-TiO_2)**	**Amplitude strain limit of the linear viscoelastic region (%)**
5-1-0	4.00
5-0-12.5	4.00
5-2-12.5	1.00
5-1-25	0.10
7-0-0	6.00
7-2-0	15.0
7-1-12.5	4.00

7-1-12.5	4.00
7-1-12.5	4.00
7-0-25	0.06
7-2-25	0.63
9-1-0	16.0
9-0-12.5	4.00
9-2-12.5	1.00
9-1-25	0.10

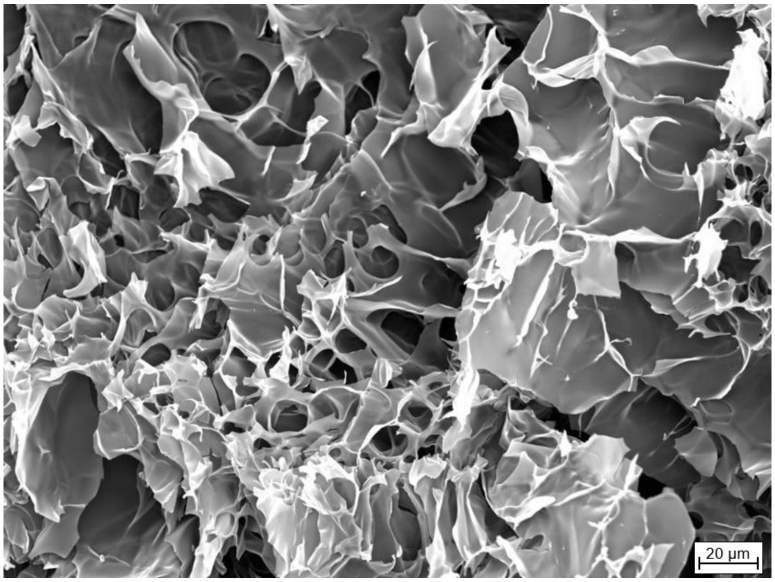

Figure S4. SEM of a CS(7)-G(0)-TiO2(0) sample after freeze drying. A three-dimensional network of CS is observed.

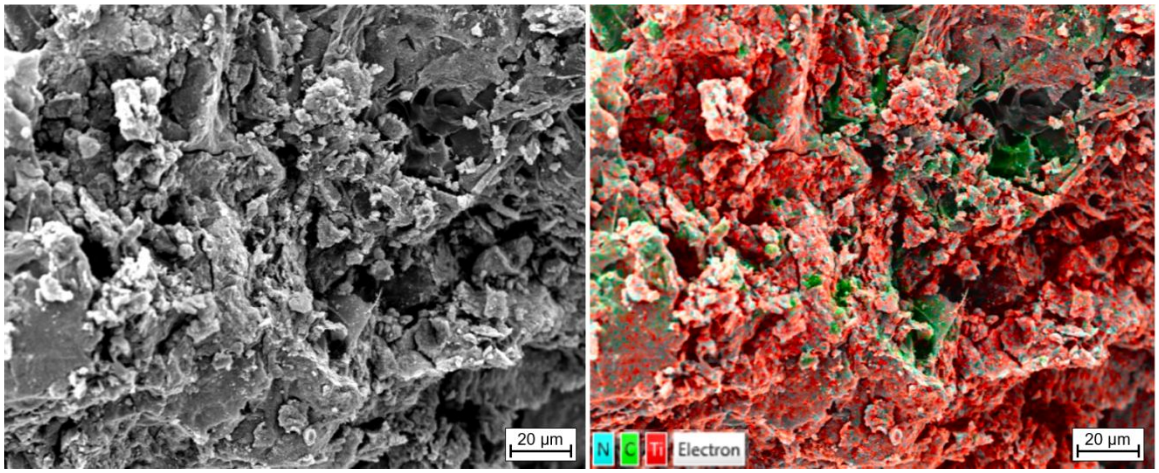

Figure S5. SEM (left) and EDS elemental mapping (right) of CS(9)-G(1)-TiO2(25) sample after freeze drying. Elemental mapping shows a homogeneous distribution of TiO2 in the material.

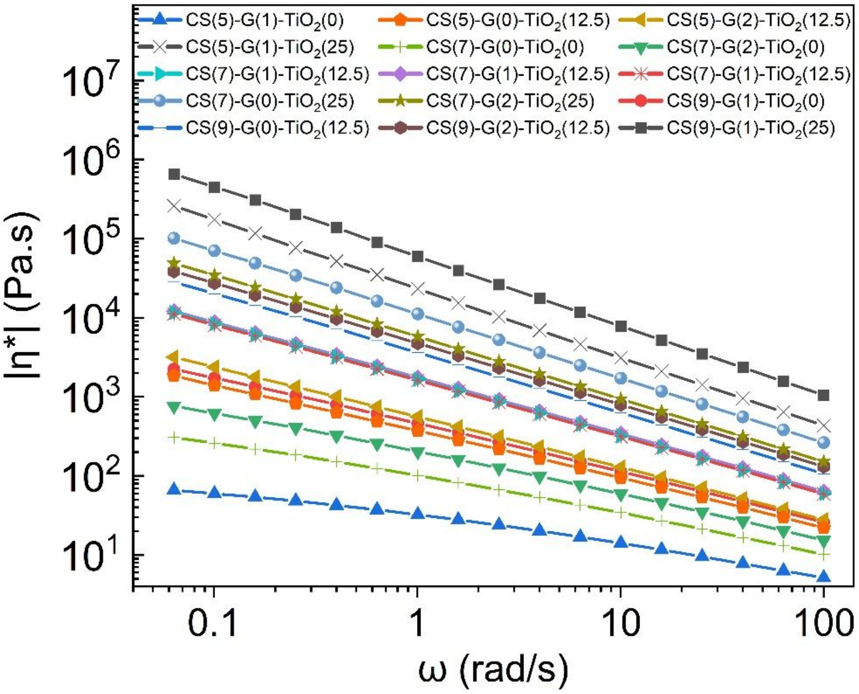

Figure S6 . Magnitude of the complex viscosity as a function of angular frequency of all 13 formulations tested.

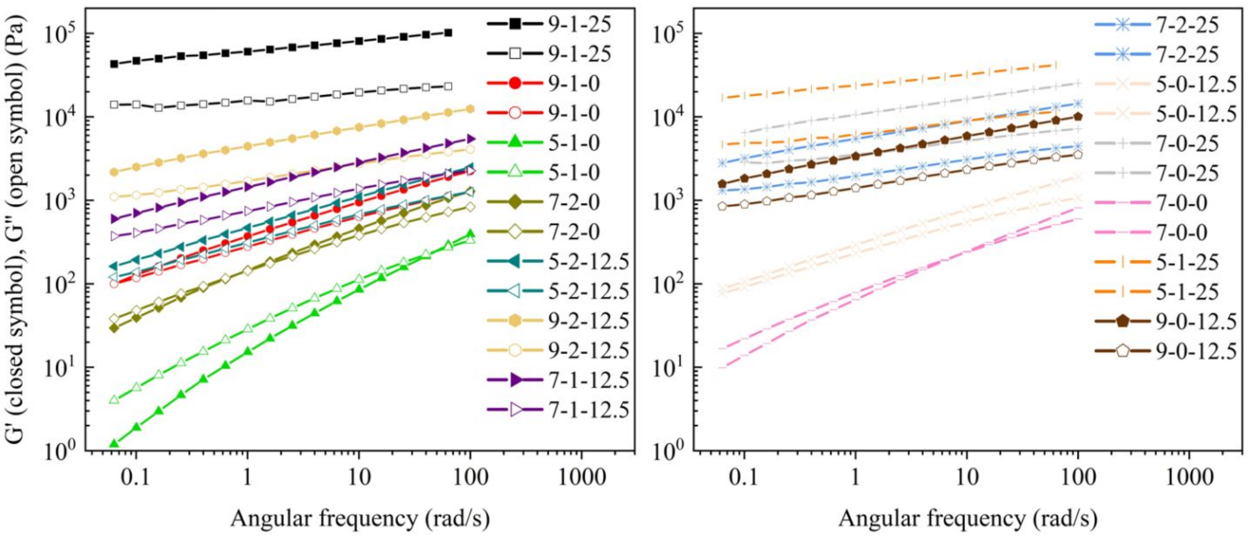

Figure S7. Frequency sweep of all formulations. Closed symbols represent storage modulus and open symbols represent loss modulus. All formulations show a frequency-dependent storage modulus.

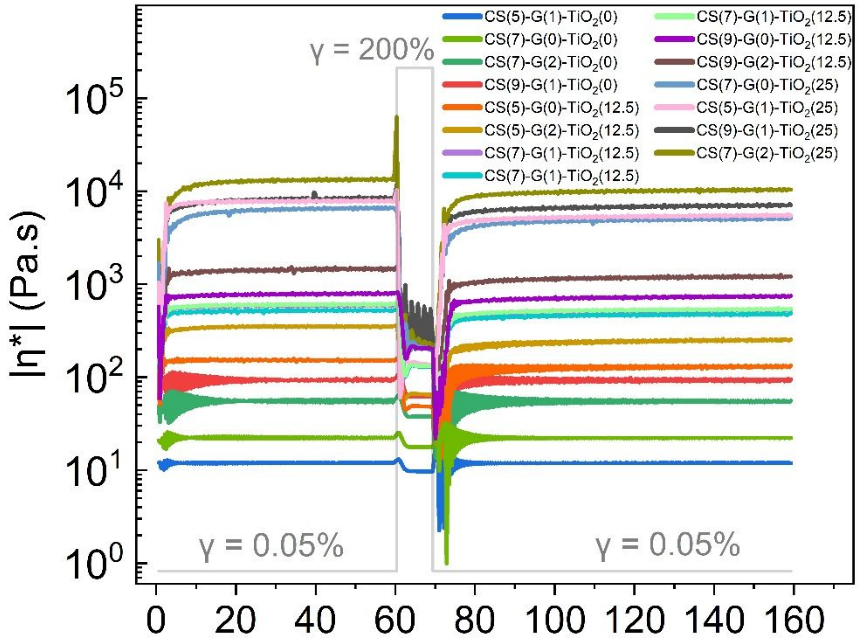

Figure S8. Magnitude of the complex viscosity during 3ITT of all DOE samples. No smoothing.

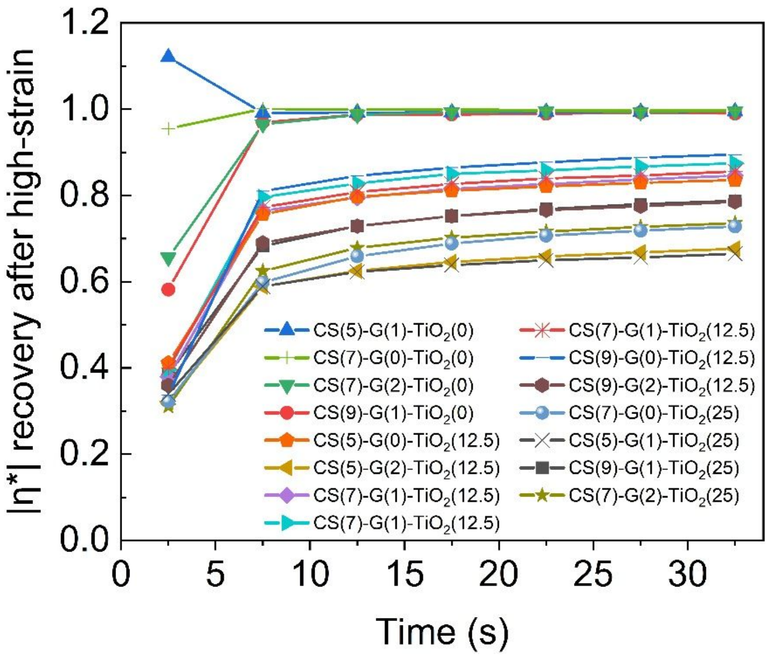

Figure S9. Magnitude of the complex viscosity recovery after high deformation interval in the 3iTT.

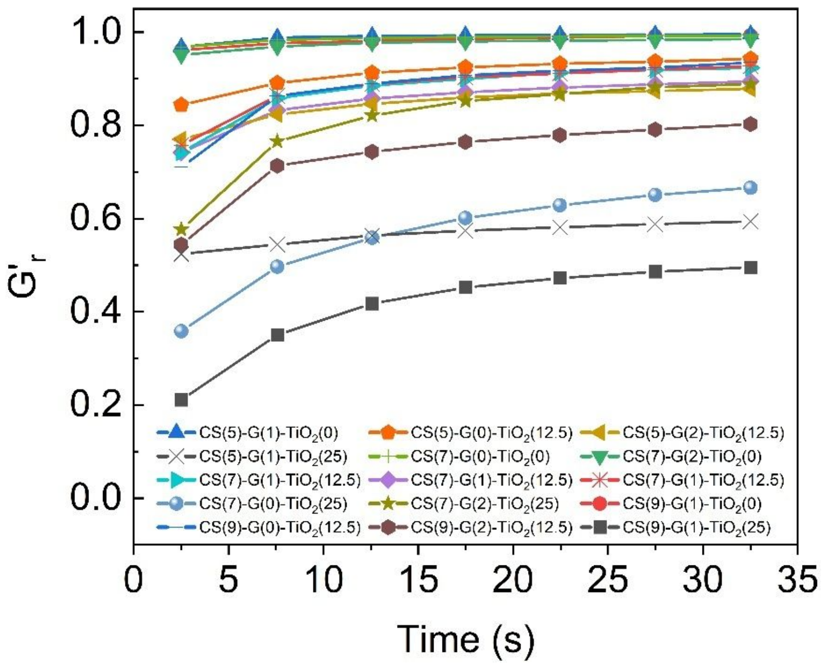

Figure S10. G' recovery after high deformation interval in 3ITTs. The result at each time point is obtained from the average of the 5 sec around it (e.g. the 2.5 s data point is the average of interval from t = 0 to t = 5 s).

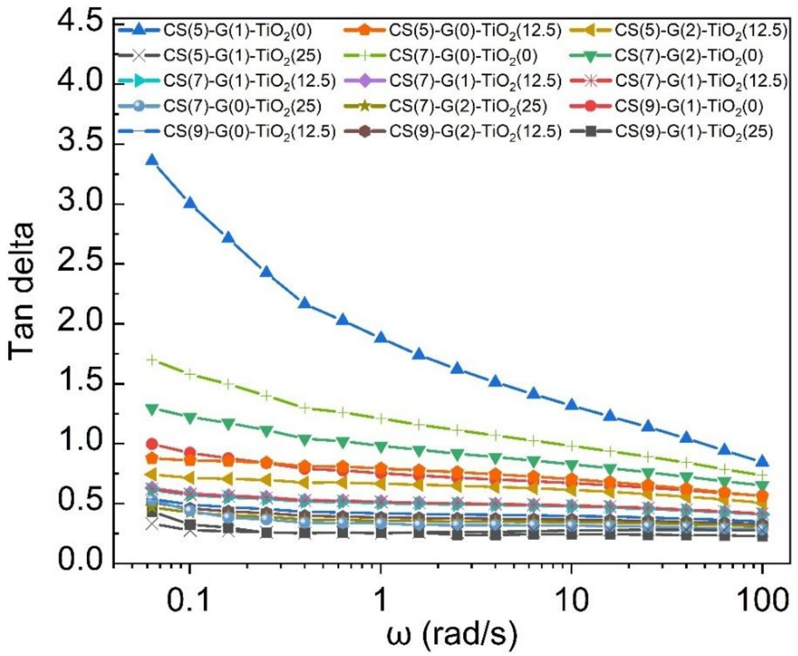

Figure S11. Tan delta as a function of angular frequency of all 13 formulations tested.

Table S2. Power law fitting ($y = b * x^a$) of tan delta as a function of angular frequency. Angular frequency values shown in this table are estimated at the transition from liquid-like to solid-like (tan delta = 1) and respective time period T ($\omega = \frac{2\pi}{T}$). 7-1-12.5 samples were prepared/tested in triplicate to assess experimental error.

**Sample (CS-G-TiO_2)**	**Power law multipler (b)**	**Power law exponent (a)**	**R^2**	**Angular frequency at tan delta = 1 (rad/s)**	**Period length required to reach tan delta = 1 (s)**
**5-1-0**	1.960	-0.180	0.992	4.2E+01	0.003778
**7-0-0**	1.239	-0.109	0.987	7.1E+00	0.022417
**7-2-0**	1.003	-0.091	0.987	1.0E+00	0.154497
**9-1-0**	0.799	-0.133	0.889	1.9E-01	0.856829
**5-0-12.5**	0.780	-0.057	0.959	1.3E-02	12.27449
**5-2-12.5**	0.664	-0.047	0.945	1.6E-04	972.0433
**9-2-12.5**	0.373	-0.097	0.931	3.9E-05	4095.039
**9-0-12.5**	0.415	-0.077	0.916	1.2E-05	13629.78
**7-1-12.5**	0.529	-0.055	0.921	9.3E-06	17050.27

**7-1-12.5**	0.532	-0.053	0.912	6.9E-06	23182.01
**7-1-12.5**	0.530	-0.050	0.898	2.9E-06	55443.66
**7-2-25**	0.350	-0.078	0.952	1.5E-06	107646
**5-1-25**	0.138	-0.133	0.945	3.5E-07	458989.6
**9-1-25**	0.224	-0.100	0.805	3.3E-07	478502.1
**7-0-25**	0.330	-0.072	0.910	2.0E-07	794652.8

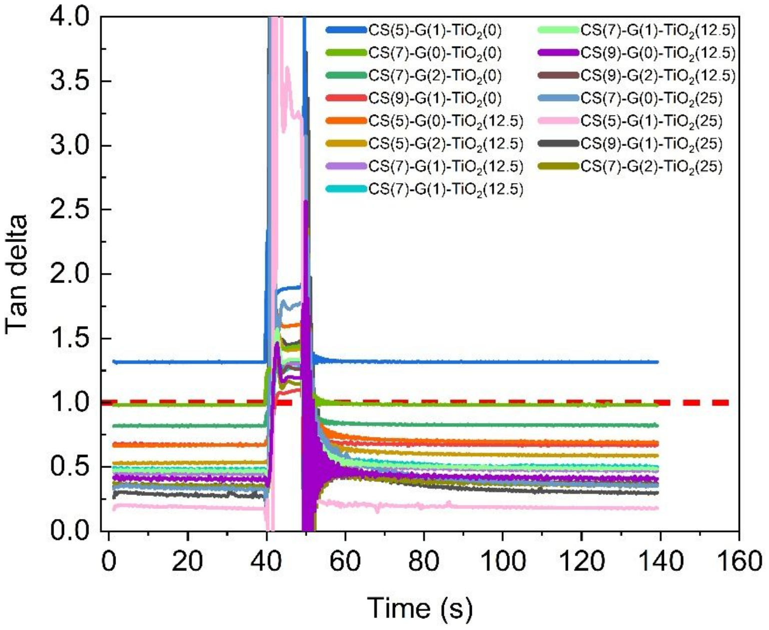

Figure S12. Tan delta values measured during 3ITT. Dashed red line indicates viscoelastic threshold from liquid-like (tan delta > 1) to solid like (tan delta < 1). All samples show a predominant liquid-like behavior during interval 2.

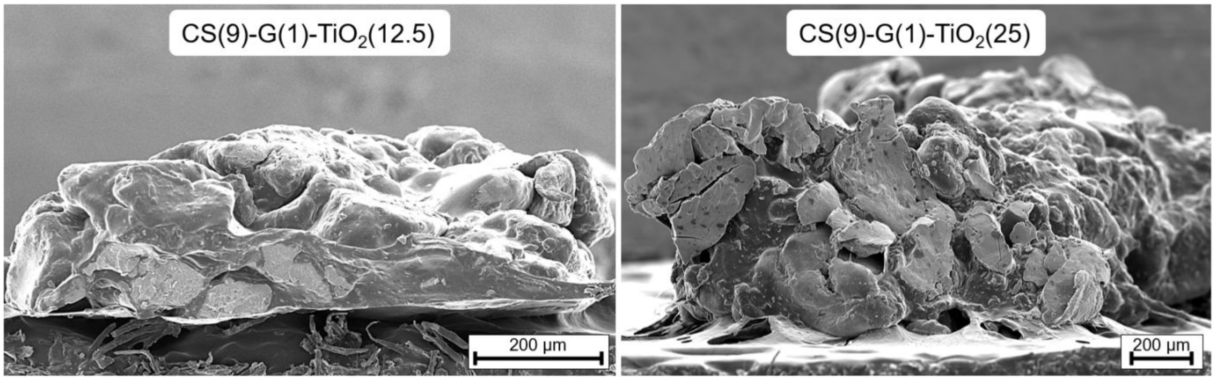

Figure S13. SEM of printed strands showing the cryofracture surface of the strand. Samples are standing on carbon tape. Larger and more frequent TiO_2 agglomerates (confirmed with EDS) are visible in the 9-1-25 sample than in the 9-1-12.5, which is likely causing the printing instability.

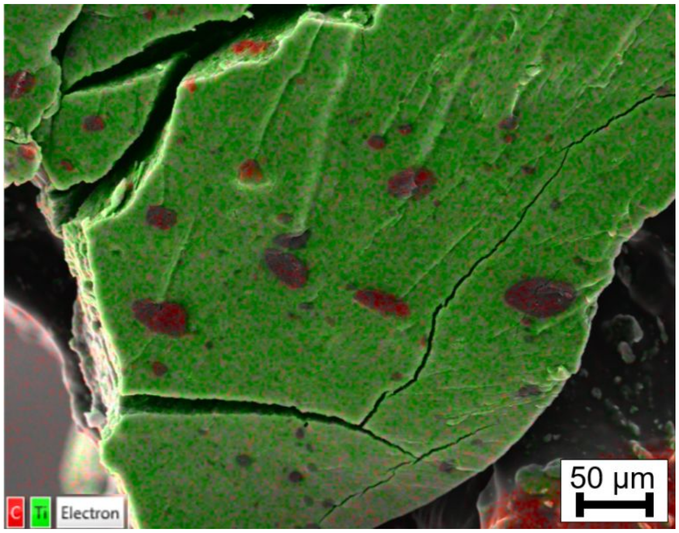

Figure S14. EDS analysis of agglomerates shown in Figure S12 confirms they are made of Ti, indicating TiO_2 agglomerates.

1. Statistical analysis

To further explore the influence of formulation components on the properties, a DOE analysis was utilized. 15 formulations (13 unique with 2 replicates of the midpoint) were studied using Minitab.

1.1 Statistical analysis of complex viscosity

Table S3. Model summary obtained from Minitab for each property fitting in the DOE.

**Property**	**S**	**R-sq**	**R-sq(adj)**	**R-sq(pred)**
Complex viscosity at ω = 0.06 rad/s	132132	79.16%	41.65%	0.00%
Complex viscosity recovery at t = 12.5 s	0.0487304	95.36%	87.01%	28.74%
Tan delta at ω = 0.06 rad/s	0.370143	91.70%	76.77%	0.00%

Complex viscosity data was analyzed at low frequencies, which better represents the longer timescales and low shear rates of the ink when deposited after going through the nozzle. The DOE analysis indicated that TiO_2 had significant influence on the magnitude of the complex viscosity of the material with a p-value of 0.036, as observed from Figure S6. CS and graphene, although influencing complex viscosity when comparing case-to-case, did not obtain statistically significant p-values from the DOE analysis. The DOE coded coefficients are displayed in Table S4**,** and the standardized effects are shown in supporting information. The model summary R^2 for the complex viscosity from the DOE fitting in Minitab was 79.16%.

Table S4. Coded coefficients for the analysis of complex viscosity at ω = 0.06 rad/s. The model only obtained statistical significance for TiO_2 (p-value < 0.05). Coefficients indicate a positive impact of CS and TiO_2 on complex viscosity.

Term	Coef	SE Coef	T-Value	P-Value	VIF
Constant	11603	76287	0.15	0.885	
**CS (%)**	**57268**	**46716**	**1.23**	**0.275**	**1**
**G (%)**	**-5003**	**46716**	**-0.11**	**0.919**	**1**
**TiO2 (%)**	**132542**	**46716**	**2.84**	**0.036**	**1**
CS (%)*CS (%)	98848	68764	1.44	0.21	1.01
G (%)*G (%)	-92525	68764	-1.35	0.236	1.01
TiO2 (%)*TiO2 (%)	118618	68764	1.73	0.145	1.01
CS (%)*G (%)	2237	66066	0.03	0.974	1
CS (%)*TiO2 (%)	98042	66066	1.48	0.198	1
G (%)*TiO2 (%)	-13141	66066	-0.2	0.85	1

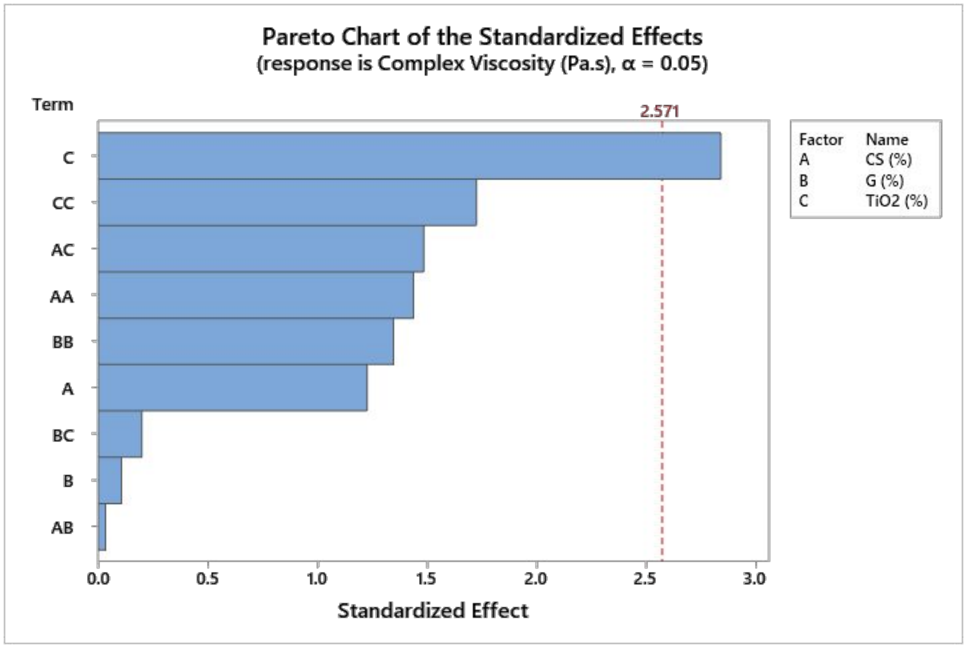

Figure S15. Standardize effects of CS, G, and TiO_2 on the magnitude of the complex viscosity at low frequency (0.04 rad/s).

The poor fitting of complex viscosity may be likely due to two reasons. First, the concentration range of CS and graphene used to make the samples were much smaller than that of TiO_2, which may have masked the statistical results. For example, increasing TiO_2 content from 0

to 25 wt% led to complex viscosity increases of hundreds to thousands of times, while increasing CS content from 5 to 9 wt% increased it by dozens of times.

Second, a case-by-case analysis of the samples showed inconsistencies in the magnitude of the complex viscosity of samples with 25 wt% of TiO_2. For example, a 5-1-25 sample displayed complex viscosity values of almost 260,000 Pa.s, while a 7-2-25 sample showed values around 49,000 Pa.s, and a 9-1-25 sample displayed values around 654,000 Pa.s. With the understanding that both CS and graphene should increase the magnitude of the complex viscosity, the complex viscosity was expected to increase from 5-1-25 to 7-2-25 to 9-1-25, which is not what is seen. These results indicate that the addition of 25 wt% of TiO_2 is reaching the mixture and dispersion limits of the procedure followed in this paper, and more studies are currently underway with different dispersion and mixture procedures.

On the other hand, for samples with a maximum of 12.5 wt% of TiO_2, the trend observed follows expected behaviors, in which increasing either CS, graphene, or TiO_2 will lead to increases in the magnitude of the complex viscosity. In these samples, TiO_2 concentration is still the most dominant factor, followed by CS, and then graphene.

As already discussed, higher complex viscosity values at low angular frequencies are desired to improve shape retention of the printed parts, which can be obtained by increasing particle content. In addition, a higher load of particles is expected to improve the efficiency of the formulations on bacteria and toxin removal from water. However, as we tailor these formulations for DIW, there is a clear tradeoff between particle content and the homogeneity of the formulations, especially considering the TiO_2 range chosen and that the procedure used is based on the work of Zetterholm et al. to prepare CS-graphene films instead of inks. Still, this research helps to better understand how particles affect CS inks for DIW, while also elucidating that 25 wt% of TiO_2 is reaching the limit of the procedure. This enables optimization of dispersion and production of inks with higher load contents.

1.2 Statistical analysis of complex viscosity recovery

A DOE analysis of complex viscosity recovery at t = 12.5 s (average result from t = 10 to 15 sec) was performed. This time was chosen because represents layer times for prints of smaller to medium parts, assessing how the complex viscosity has recovery when a new layer is deposited. For larger parts, longer layer times are expected, where the complex viscosity recovery would be

even higher. The DOE analysis for the complex viscosity recovery at t = 12.5 s resulted in an R^2 of 95.4%. The coded coefficients are shown in Table S5.

Table S5. Coded coefficients for the analysis of complex viscosity recovery at t = 12.5. Negative coefficients of TiO_2 and graphene indicate diminished recovery caused by these components, while a positive coefficient of CS indicates facilitated recovery. P-values indicate a statistically significance response for TiO_2, but significance levels for CS and graphene are higher than 0.05.

**Term**	**Coef**	**SE Coef**	**T-Value**	**P-Value**	**VIF**
Constant	0.810	0.0281	28.8	0	
**CS (%)**	**0.032**	**0.0172**	**1.84**	**0.125**	**1**
**G (%)**	**-0.035**	**0.0172**	**-2.04**	**0.097**	**1**
**TiO2 (%)**	**-0.160**	**0.0172**	**-9.26**	**0.000**	**1**
CS (%)*CS (%)	-0.030	0.0254	-1.17	0.296	1.01
G (%)*G (%)	-0.032	0.0254	-1.25	0.268	1.01
TiO2 (%)*TiO2 (%)	0.052	0.0254	2.06	0.095	1.01
CS (%)*G (%)	0.014	0.0244	0.56	0.597	1
CS (%)*TiO2 (%)	0.028	0.0244	1.14	0.306	1
G (%)*TiO2 (%)	0.008	0.0244	0.33	0.758	1

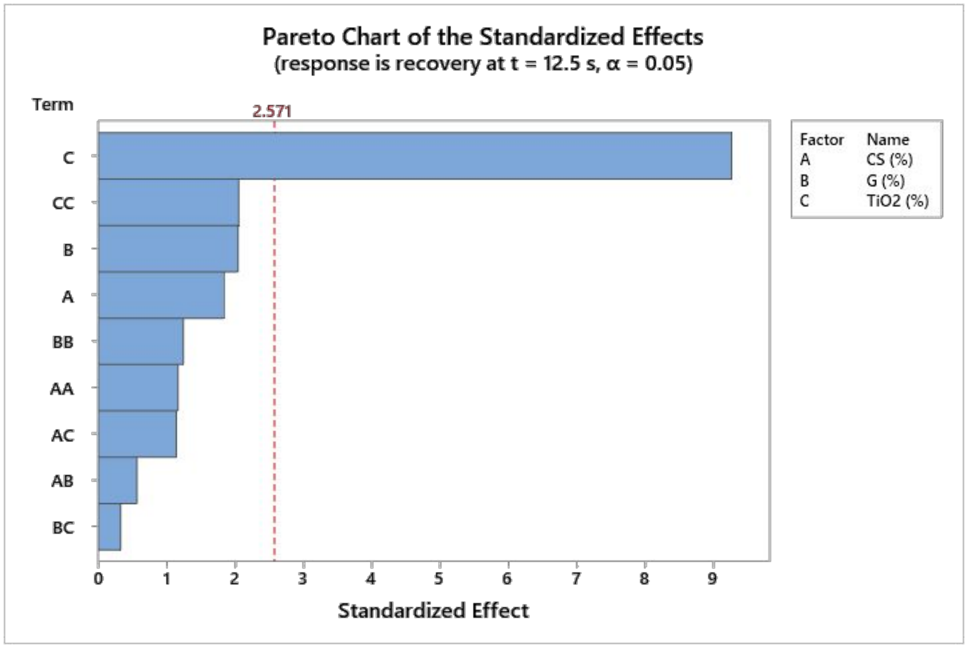

Figure S16. Standardize effects of CS, G, and TiO_2 on the complex viscosity recovery at 12.5 sec after high deformation.

The DOE analysis indicates that TiO_2 is the component affecting the complex viscosity recovery after deformation at t = 12.5 s the most. The negative coefficient observed from it shows that increases in TiO_2 concentration lead to decreases in recovery, as was observed in the case-by-case analysis in Figure S8. Following the same trend observed with the complex viscosity analysis, the much higher loads of TiO_2 will limit the DOE model to observe the small variations in properties caused by changes in CS and graphene, leading to a type II statistical error. Even though CS and graphene are both contributing to the recovery behavior of the material from direct comparison of formulations, the DOE analysis indicated a statistically insignificant contribution of them to the system. The coefficient analysis for CS and graphene indicates that CS leads to more recovery, while graphene decreases the recovery of the system.

1.3 Statistical analysis of tan delta

The DOE analysis for tan delta was performed at low angular frequencies, which are more representative of printed material at rest (longer observation times). The DOE analysis of tan delta at ω = 0.06 rad/s resulted in a R^2 of 91.7%. Standardized effects are shown in Table S6.

Table S6. Coded coefficients for the analysis of tan delta at ω = 0.06 rad/s. Coefficients have negative values for CS, graphene, and TiO_2 in the tan delta value. P-values indicates a statistically significance response for CS and TiO_2, but significance levels for graphene are higher than 0.05.

Term	Coef	SE Coef	T-Value	P-Value	VIF
Constant	0.622	0.214	2.91	0.033	
**CS (%)**	**-0.355**	**0.131**	**-2.71**	**0.042**	**1**
**G (%)**	**-0.079**	**0.131**	**-0.6**	**0.575**	**1**
**TiO2 (%)**	**-0.698**	**0.131**	**-5.34**	**0.003**	**1**
CS (%)*CS (%)	0.163	0.193	0.85	0.435	1.01
G (%)*G (%)	-0.118	0.193	-0.61	0.566	1.01
TiO2 (%)*TiO2 (%)	0.495	0.193	2.57	0.05	1.01
CS (%)*G (%)	0.026	0.185	0.14	0.892	1
CS (%)*TiO2 (%)	0.616	0.185	3.33	0.021	1
G (%)*TiO2 (%)	0.086	0.185	0.46	0.663	1

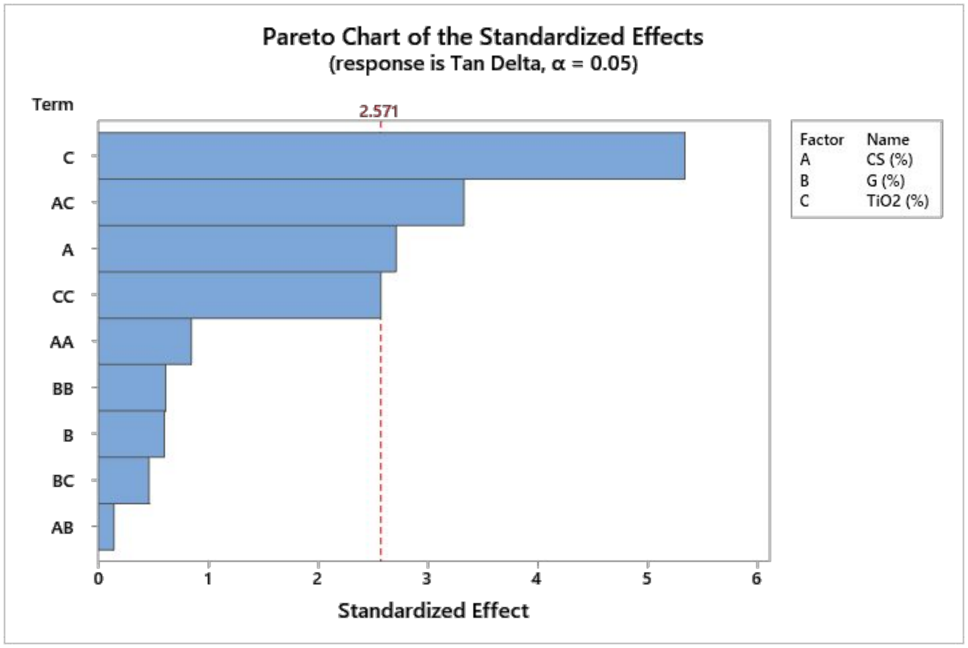

Figure S17. Standardize effects of CS, G, and TiO_2 on the tan delta values at ω = 0.06 rad/s. Both TiO_2 (p-value = 0.003) and CS (p-value = 0.042) are statistically significant.

In agreement with the data shown in Figure S10, the surface response of the design shows that TiO_2 was the factor contributing the most to decreases in tan delta, and the model also found CS as a contributing factor, even if to a smaller extent. On the other hand, the design indicated that graphene did not have a statistically significant influence on the tan delta values at low frequencies. Similar to the observations made to the complex viscosity section, a case-by-case analysis shows some influence of the presence of graphene in the viscoelastic behavior of the formulations (Figure S17), but this response is much less pronounced than that of TiO_2 and CS, leading to the model likely not being able to identify it. Coefficients indicate that all three components in the formulation are negative, indicating that they decrease tan delta values, leading to a more elastic response, which is desired for DIW. TiO_2 is the component impacting it the most with a coefficient of -0.698.

1.4 Response optimization from statistical model

A response optimization model was applied to the data in Minitab to obtain a formulation with maximized complex viscosity at ω = 0.06 rad/s and complex viscosity recovery at t = 12.5 s, while minimizing tan delta at ω = 0.06 rad/s and axial stress at ω = 100 rad/s. These parameters are believed to facilitate and improve printability in DIW. Table S7 shows the results of the DOE response optimization for the given conditions.

Table S7. DOE Response optimization for a formulation maximizing complex viscosity and complex viscosity recovery at t = 12.5 and minimizing tan delta.

**Formulation**			**Data fit**			
**CS (%)**	**G (%)**	**TiO_2 (%)**	**Complex Viscosity (Pa.s)**	**Recovery at t = 12.5 s**	**Tan Delta**	**Composite Desirability**
9	0.83	13.89	192,802	0.82	0.43	0.56

The model chose the maximum amount of CS available, which makes sense, considering that increasing CS leads to increases in complex viscosity and complex viscosity recovery, while also facilitating decreases in tan delta; these characteristics are typically desired for DIW.

Regarding graphene, the model chose a value slightly below the midpoint of the ranges available, at 0.83 wt%. However, for many of the properties studied, the effect of graphene was irrelevant, and the model could not capture its effects. Graphene leads to slight increases in complex viscosity and decreases in tan delta, which are important. However, one of the pronounced effects of graphene was on the complex viscosity recovery at t = 12.5 s, where increases in graphene concentration led to smaller recoveries when in combination with TiO_2. Therefore, limiting the amount of graphene in the system to the midpoint formulation is beneficial, while optimizing its usefulness. Even if graphene had no impact on the rheological properties and printability of CS-G-TiO_2 inks, its addition to the ink is still important when thinking about water remediation applications. Lastly, TiO_2 was chosen at 13.89 wt%, slightly above the midpoint of the available range. Increasing TiO_2 content results in remarkable increases in complex viscosity and decreases in tan delta, but at the cost of decreasing complex viscosity recovery. The choice of 13.89 wt% is also viable considering the challenges in making CS-G-TiO_2 inks with 25 wt% of TiO_2, which leads to more inconsistencies in the properties. Figure S18 illustrates the curves utilized by Minitab for the optimization of the rheological response of CS-G-TiO_2 inks, indicating how the model expects each component in the formulation to impact rheological properties.

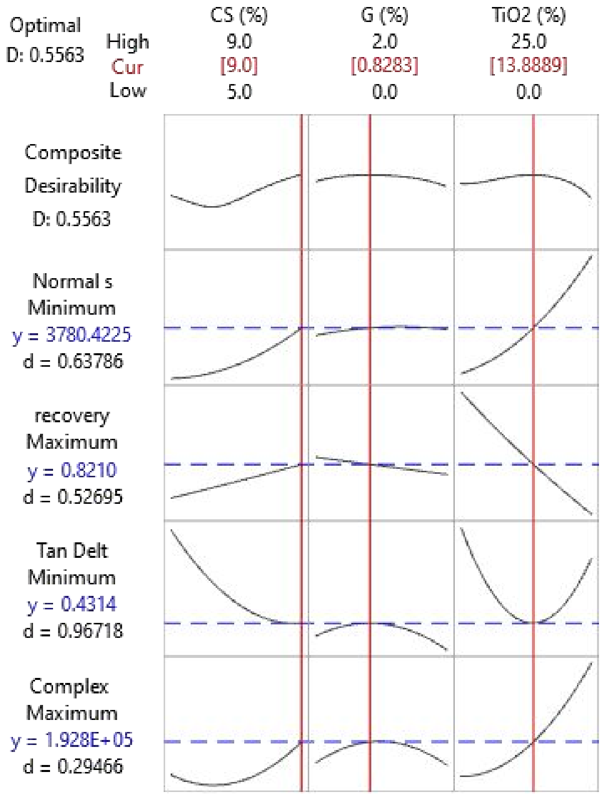

Figure S18. Optimization curves from DOE response optimization. The curves indicate the model expected rheological response for CS, graphene, and TiO_2. (Top to bottom: composite desirability, axial stress at ω = 100 rad/s, complex viscosity recovery at t = 12.5 s, tan delta at ω = 0.06 rad/s, and complex viscosity at ω = 0.06 rad/s)

Agreeing with the statistical results previously discussed, the accuracy level of the model varies depending on the property and component analyzed. For CS, even though the p-values obtained are not always significant, the model response matches the response observed in the case-by-case analysis of the formulations. Increases in CS content leads to an ink behavior better suited for DIW.

For graphene, the model captured very little of its influence on the properties of the inks, which agrees with the experimental observations, as in the case of tan delta. However, the model fails to detect the influence, even if small, of graphene on the complex viscosity and complex viscosity recovery.

When analyzing TiO_2 response, which displayed statistically significant p-values for all four properties, the model matches the experimental response well for all properties other than tan delta,

in which it expects increases in tan delta at low and high TiO_2 contents, which is not what was observed experimentally, where tan delta decreased with increasing TiO_2 content. The impact of TiO_2 was the most prominent from all three components studied, especially because the ranges utilized were larger (up to 25 wt%) to maximize water remediation properties.
